# Pentraxin 3 regulated by miR-224-5p modulates macrophage reprogramming and exacerbates osteoarthritis associated synovitis by targeting CD32

**DOI:** 10.1038/s41419-022-04962-y

**Published:** 2022-06-24

**Authors:** Jianbin Yin, Hua Zeng, Kai Fan, Haoyu Xie, Yan Shao, Yuheng Lu, Jinjian Zhu, Zihao Yao, Liangliang Liu, Hongbo Zhang, Bingsheng Luo, Xinjie Wang, Chun Zeng, Xiaochun Bai, Haiyan Zhang, Daozhang Cai

**Affiliations:** 1 Department of Joint Surgery, Center for Orthopaedic Surgery, The Third Affiliated Hospital of Southern Medical University, Guangzhou, China; 2 Department of Orthopedics, Orthopedic Hospital of Guangdong Province, Academy of Orthopedics·Guangdong Province, The Third Affiliated Hospital of Southern Medical University, Guangzhou, China; 3 The Third School of Clinical Medicine, Southern Medical University, Guangzhou, China; 4 Guangdong Provincial Key Laboratory of Bone and Joint Degeneration Diseases, Guangzhou, China

**Keywords:** Osteoarthritis, Extracellular signalling molecules

## Abstract

Emerging evidence has shown an imbalance in M1/M2 macrophage polarization to play an essential role in osteoarthritis (OA) progression. However, the underlying mechanistic basis for this polarization is unknown. RNA sequencing of OA M1-polarized macrophages found highly expressed levels of pentraxin 3 (PTX3), suggesting a role for PTX3 in OA occurrence and development. Herein, PTX3 was found to be increased in the synovium and articular cartilage of OA patients and OA mice. Intra-articular injection of PTX3 aggravated, while PTX3 neutralization reversed synovitis and cartilage degeneration. No metabolic disorder or proteoglycan loss were observed in cartilage explants when treated with PTX3 alone. However, cartilage explants exhibited an OA phenotype when treated with culture supernatants of macrophages stimulated with PTX3, suggesting that PTX3 did not have a direct effect on chondrocytes. Therefore, the OA anti-chondrogenic effects of PTX3 are primarily mediated through macrophages. Mechanistically, PTX3 was upregulated by miR-224-5p deficiency, which activated the p65/NF-κB pathway to promote M1 macrophage polarization by targeting CD32. CD32 was expressed by macrophages, that when stimulated with PTX3, secreted abundant pro-inflammation cytokines that induced severe articular cartilage damage. The paracrine interaction between macrophages and chondrocytes produced a feedback loop that enhanced synovitis and cartilage damage. The findings of this study identified a functional pathway important to OA development. Blockade of this pathway and PTX3 may prevent and treat OA.

## Introduction

Osteoarthritis (OA) is the most common form of arthritis, with knee and hip joints the primary sites of involvement [1]. OA is related to a variety of factors including age, excessive stress, sex, and obesity [2–4]. The main pathological features of OA are destruction of articular cartilage, sclerosis of subchondral bone, formation of osteophytes, synovitis, and ligament degradation, which lead to pain and dysfunction of the affected joints [5]. With the progression of OA, patients lose motor function and become disabled, which decreases the quality of life and increases the risk for early death [1, 6].

Recent studies have shown that synovitis during the early stage of articular cartilage destruction increases OA risk [7, 8]. Acute, subacute, and chronic injuries initiate destruction of articular cartilage. Products of cartilage destruction induce local inflammation of the synovium, promoting cartilage destruction, more inflammation, and a vicious disease cycle [9–11]. Intra-articular inflammation is the primary driver of OA cartilage destruction and OA progression.

Polarized macrophages play an important role in the development of synovitis. Synovitis is a precursor to OA and macrophages are central to the pathological process [12]. In a rat model of disease, SPECT-CT imaging technology has demonstrated macrophage activation throughout OA progression, in particular during early disease stages [13]. M1-polarized macrophages produce a large number of pro-inflammatory cytokines including; IL-1β, IL-6, IL-12, and TNF-α [14]. In contrast, M2-polarized macrophages are anti-inflammatory, secreting IL-10 and IL-4, as well as playing a role in tissue repair [15, 16]. As such, activated macrophages have important roles in the progression of OA. Our previous studies have shown that M1 polarization of synovial macrophages aggravates collagenase-induced experimental OA, while M2-polarization reduces the development of OA [17].

Pentraxin 3 (PTX3), an acute inflammatory protein, is primarily secreted by neutrophils, macrophages, and other immune cells [18–21]. PTX3 plays an important role in inflammation and in diseases such as rheumatoid arthritis, microbial infection, and atherosclerosis [22–24]. Increased levels of gastric cancer associated PTX3 promote macrophage recruitment and cancer-related inflammation [25]. After knockout of PTX3 in a variety of animal cancer models, expression levels of M2 macrophage related genes are upregulated [18]. In another study, the PTX3 gene family was shown to regulate macrophage physiology and pathology [26]. These results suggest that PTX3 is related to the polarization of macrophages. Since disease associated macrophages secrete PTX3 [27], it is possible that PTX3 has a similar role in OA. No reports have associated PTX3 with OA, and there is no description of a specific mechanism by which PTX3 promotes macrophage polarization.

In this study, a DMM (destabilization of the medial meniscus)-OA mouse model and the cell line, RAW264.7, were used to examine the interaction of PTX3 with macrophages and the contribution of this interaction to OA. We found PTX3 levels and the activation of the NF-κB pathway to be significantly upregulated in OA, with the activation due to CD32 targeting. Pathway activation promoted M1 polarization of OA synovial macrophages, which aggravated OA. Increased expression of PTX3 was related to downregulation of miR-224-5p. Further, a cyclical PTX3-macrophage-chondrocyte model is described, which provides a means by which understand the mechanistic basis of OA occurrence and development. This model will allow for the exploration of novel targets for OA treatment.

## Results

### Increased expression of PTX3 in the synovium and articular cartilage of OA patients and OA mice

**Fig. 1:**
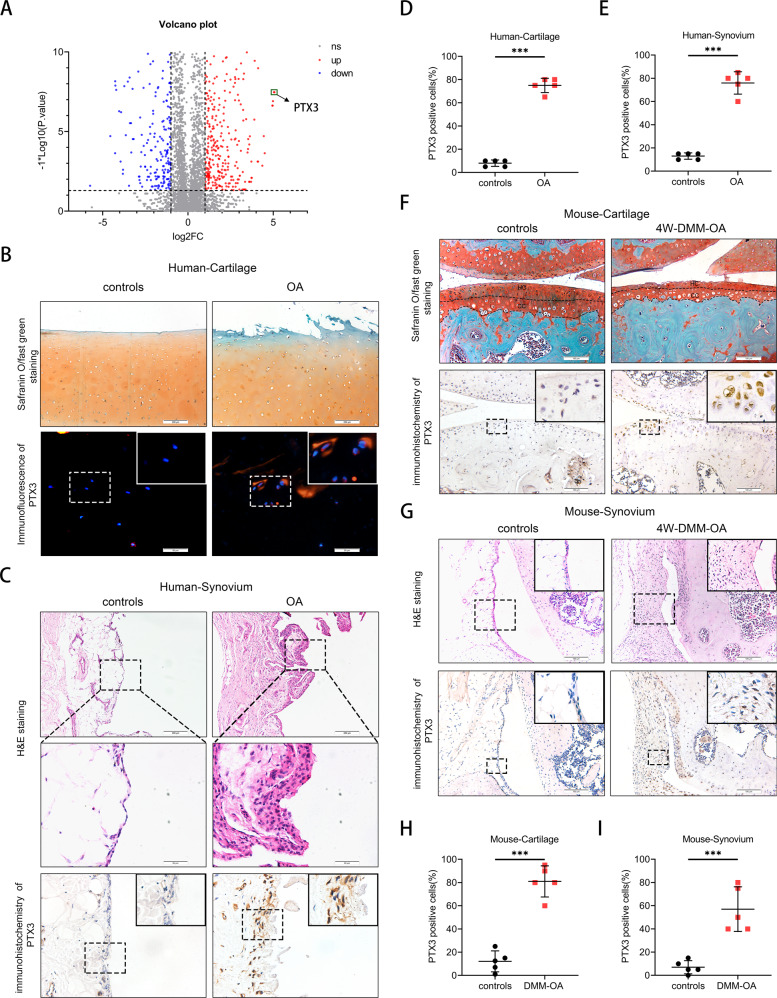
Increased expression of PTX3 in the synovium and articular cartilage of OA patients and OA mice. **A** Differentially expressed mRNAs in macrophages from TSC1KO and WT mice [17]. **B** Safranin O/fast green staining (upper) and immunofluorescence (IF) of PTX3 (lower) of human knee cartilage from the medial (OA) and lateral (controls) tibial plateau of OA patients. Scale bar: 50 µm, 200 µm. **C** Hematoxylin and eosin (H&E) (upper) and immunohistochemistry (IHC) of PTX3 (lower) of human synovial tissues from patients with no history of arthritis and OA patients. Scale bar: 50 µm, 200 µm. **D** Quantification of PTX3 in human knee cartilage of the medial and lateral tibial plateau described in **B**, *n* = 5 per group. **E** Quantification of PTX3 in human synovium described in **C**, *n* = 5 per group. **F** Safranin O/fast green staining (upper) and IHC of PTX3 (lower) of knee cartilage from controls and OA mice at 8 weeks post destabilization of the medial meniscus (DMM). Scale bar: 100 µm. **G** H&E (upper) and IHC of PTX3 (lower) of synovial tissues from controls and 8 weeks old-DMM-OA mice. Scale bar: 100 µm. **H** Quantification of PTX3 in knee cartilage described in (**F**), *n* = 5 per group. **I** Quantification of PTX3 in synovial tissues described in **G**, *n* = 5 per group. *P < 0.05, **P < 0.01, ***P < 0.001, ns not significant. Data are shown as means ± SD. Statistical analyses were conducted by unpaired *t*-test. Boxed area is enlarged in the top right corner.

We previously demonstrated an essential role for enhanced M1 macrophage involvement in the progression of OA [17]. However, the underlying mechanistic basis for the involvement of macrophages was not identified. Herein, mRNA expression profiles of M1 macrophages and control macrophages were analyzed (RNA sequencing data have been deposited in the NCBI SRA database under accession codes SRR6660735 and SRR6660734) [17]. By performing Gene Ontology (GO) and Kyoto Encyclopedia of Genes and Genomes (KEGG) analysis, we found PTX3 to be one of the most highly upregulated genes of M1 macrophages (Fig. 1A).

To explore expression of PTX3 during the occurrence and development of OA, synovium and cartilage from OA patients and OA mice were collected. Immunohistochemistry (IHC) and immunofluorescence (IF) staining showed a marked increase in PTX3 in OA synovial tissue and articular cartilage, which was associated with synovitis and cartilage damage (Fig. 1B–E). Enzyme-linked immunosorbent assay (ELISA) demonstrated high levels of PTX3 in the serum and synovial fluid of OA patients in comparison to controls (Supplemental Fig. 1A, B). Similarly, increased expression of PTX3 was found in the DMM-OA mouse model (Fig. 1F–I, Supplemental Fig. 1C). PTX3 expression was significantly upregulated in both LPS-treated RAW264.7 cell and LPS- or IL-1β-treated mouse primary chondrocytes (Supplementary Fig. 1D, E). Taken together, these observations demonstrate upregulation of PTX3 by M1 macrophages, and increased PTX3 levels in the synovium and articular cartilage of OA patients and OA mice. These results suggest associations among PTX3, synovitis, and cartilage damage during OA development.

### PTX3 accelerates synovitis, cartilage erosion, and OA exacerbation in mice

**Fig. 2:**
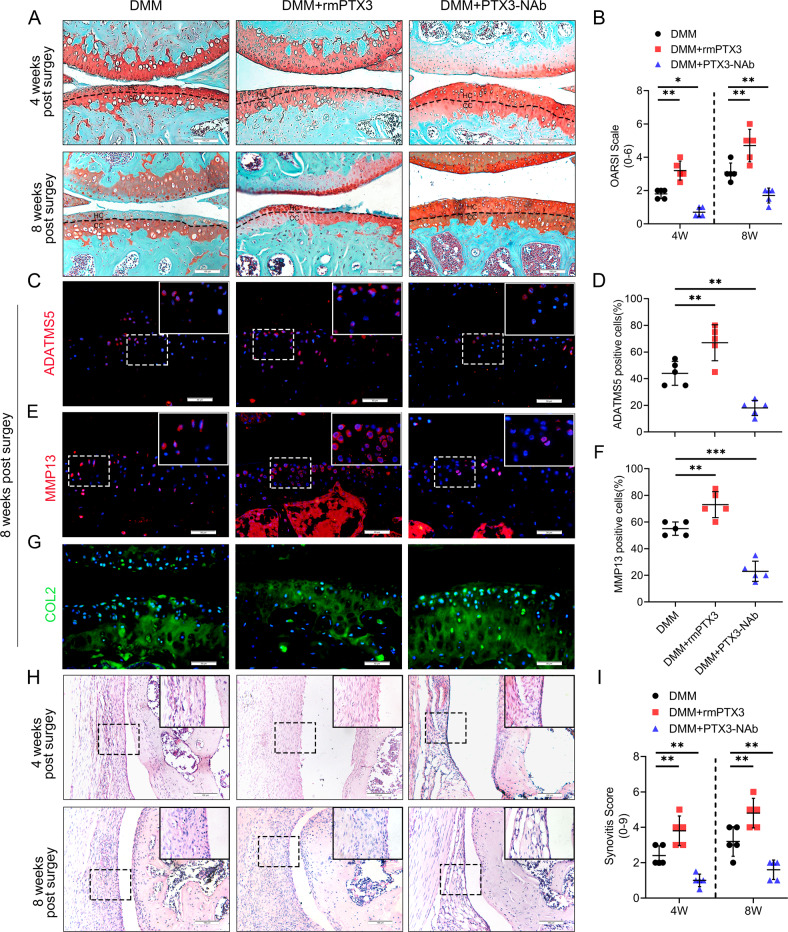
PTX3 accelerates synovitis, cartilage erosion, and exacerbates OA development in mice. **A**, **B** Safranin O/fast green staining and Osteoarthritis Research Society International (OARSI) grades of knee cartilage from DMM mice treated with vehicle, recombinant mouse PTX3 (rmPTX3), or PTX3 neutralizing antibody (PTX3-NAb) for 4 weeks and 8 weeks. Scale bar: 100 µm. **C**–**F** IF staining and quantification of ADATMS5 and MMP13 in knee cartilage of controls and DMM mice treated with vehicle, rmPTX3, or PTX3-NAb for 8 weeks. Scale bar: 50 µm. **G** IF staining and quantification of COL2 in knee cartilage of controls and DMM mice treated with rmPTX3 or PTX3-NAb for 8 weeks. Scale bar: 50 µm. **H**, **I** H&E and synovitis score of synovial tissues from DMM mice treated with vehicle, rmPTX3, or PTX3-NAb for 4 weeks and 8 weeks. Scale bar: 100 µm. *P < 0.05, **P < 0.01, ***P < 0.001, ns not significant. Data are shown as means ± SD. Statistical analyses were conducted by two-way analysis of variance followed by Sidak’s multiple comparison test (**B** and **I**) or one-way analysis of variance followed by Dunnett’s multiple comparison test (**D** and **F**). Boxed area is enlarged in the top right corner.

To assess the role of PTX3 in OA, 10-week-old male C57 mice were administered an intra-articular injection of PTX3 recombinant protein or neutralizing antibody once per week following DMM surgery. Exogenous PTX3 accelerated OA development in mice characterized by: fewer chondrocytes; greater cartilage destruction and proteoglycan loss; decreased COL2, SOX9, ACAN expression; and increased MMP13, ADAMTS5, and COLX expression in tibial cartilage. Results were confirmed by the Osteoarthritis Research Society International (OARSI) scale at both 4 weeks and 8 weeks post DMM surgery (Fig. 2A–G and Supplementary Fig. 2A–D). Compared to controls, high levels of synovial hyperplasia and abundant cell infiltration were observed in the synovial tissue of OA mice treated with PTX3 recombinant protein, as well as significantly higher synovitis scores post DMM surgery (Fig. 2H, I). Moreover, synovitis and cartilage erosion were prevented by PTX3 neutralizing antibody, with the upregulation of COL2, SOX9, and ACAN expression. Further, MMP13, ADAMTS5, and COLX were inhibited, demonstrating that reduced PTX3 can rescue the catabolism of chondrocytes and synovial inflammation during OA development (Fig. 2A–I and Supplementary Fig. 2A–G). These findings suggest that PTX3 plays an essential role in OA pathology and that PTX3 supplementation aggravates, while PTX3 neutralization reverses, synovitis and cartilage degeneration.

### PTX3 enhances M1 polarization and inflammatory cytokine production within OA synovium

**Fig. 3:**
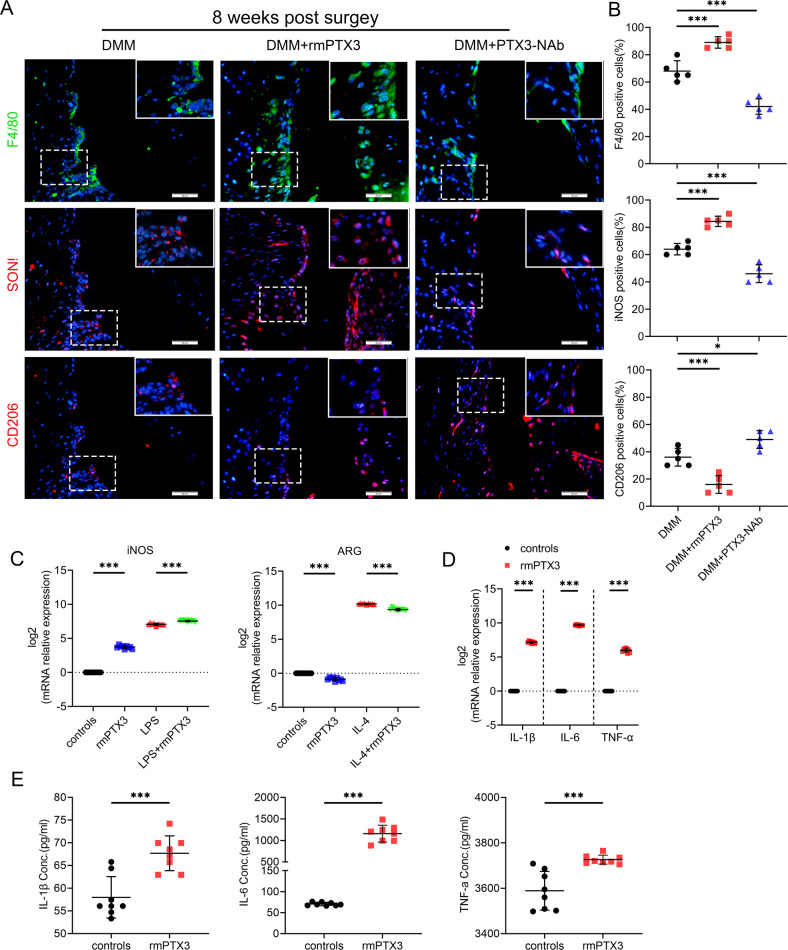
PTX3 enhances M1 polarization and promotes production of inflammatory cytokines in OA synovium. **A**, **B** IF staining and quantification of F4/80 (upper), iNOS (middle), and CD206 (lower) in synovial tissues of DMM mice treated with vehicle, rmPTX3 or PTX3-NAb for 8 weeks, *n* = 5 per group. Scale bar: 50 µm. **C** Relative mRNA expression level of iNOS and ARG in LPS-, IL-4- or rmPTX3-treated RAW264.7 cells, *n* = 9 per group. **D** Relative mRNA expression level of IL-1β, IL-6, and TNF-α in rmPTX3-treated RAW264.7 cells, *n* = 9 per group. **E** ELISA of IL-1β, IL-6, and TNF-α in the supernatants of RAW264.7 treated with rmPTX3, *n* = 8 per group. *P < 0.05, **P < 0.01, ***P < 0.001, ns not significant. Data are shown as means ± SD. Statistical analyses were conducted by one-way analysis of variance followed by Dunnett’s multiple comparison test (**B**), two-way analysis of variance followed by Sidak’s multiple comparison test (**C**) or unpaired *t*-test (**D** and **E**). Boxed area is enlarged in the top right corner.

We assessed whether PTX3 modulated macrophage polarization and synovitis. Compared with controls, a marked elevation in F4/80 (macrophage marker)-positive cells was detected in the synovial tissues of recombinant mouse (rmPTX3)-treated OA mice. There was also a significant increase in iNOS (M1-like macrophage marker) and a decrease in CD206 (M2-like macrophage marker)-positive cells. With administration of PTX3 neutralizing antibody, F4/80 and iNOS were downregulated and CD206 was upregulated in mouse synovium (Fig. 3A, B). Similar findings were observed with LPS or IL-4 stimulated RAW264.7 cells (Fig. 3C and Supplementary Fig. 3A, B). Moreover, stimulation with PTX3 significantly promoted the expression of proinflammatory IL-1β, IL-6, and TNF-α by RAW264.7 cells (Fig. 3D). IL-1β, IL-6, and TNF-α were significantly increased in the supernatants of rmPTX3-stimulated RAW264.7 cells, as judged by ELISA (Fig. 3E). Taken together, these results suggest a role for PTX3 in the reprogramming of macrophages and OA synovitis.

### PTX3 indirectly affects chondrocyte metabolism by regulation of macrophage polarization

**Fig. 4:**
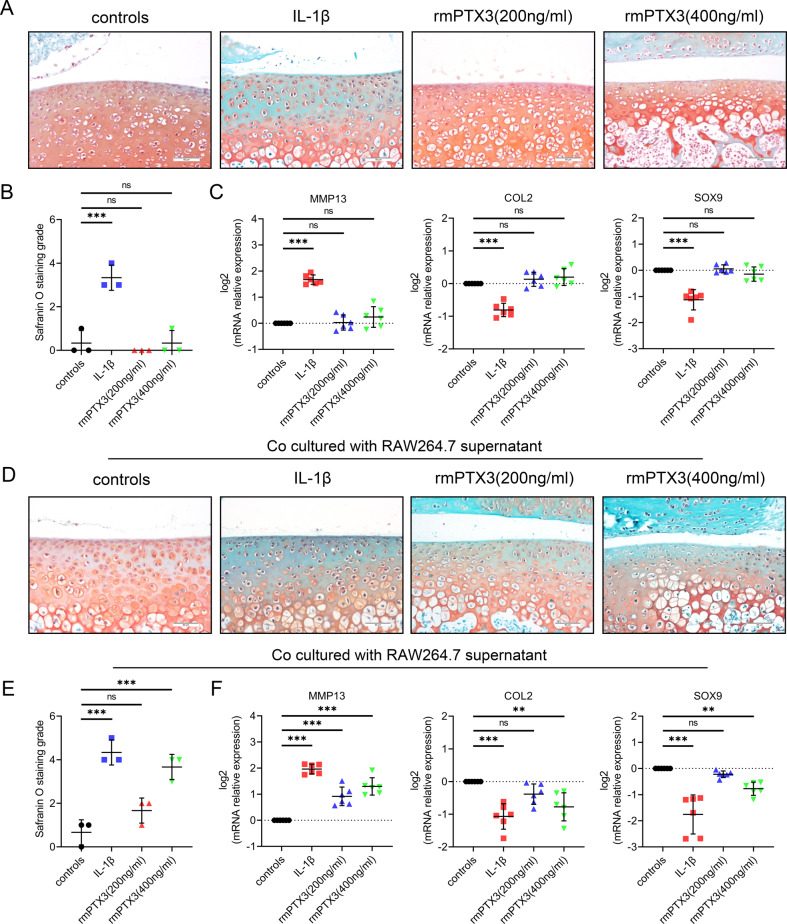
PTX3 has indirect effects on chondrocyte metabolism through regulation of macrophage polarization. **A**, **B** Safranin O staining and grade of mouse tibial plateaus cartilage explants treated with IL-1β or rmPTX3, *n* = 3 per group; Scale bar: 50 µm. **C** Relative mRNA expression levels of MMP13, COL2, and SOX9 in IL-1β- or rmPTX3-treated primary murine chondrocytes, *n* = 6 per group. **D**, **E** Safranin O staining and grade of mouse tibial plateaus cartilage explants co-cultured with supernatants of RAW264.7 (RAW264.7 was treated with IL-1β and rmPTX3), *n* = 3 per group; Scale bar: 50 µm. **F** Relative mRNA expression levels of MMP13, COL2 and SOX9 in primary murine chondrocytes co-cultured with supernatants of RAW264.7 (RAW264.7 was treated with IL-1β or rmPTX3), *n* = 6 per group. *P < 0.05, **P < 0.01, ***P < 0.001, ns not significant. Data are shown as means ± SD. Statistical analyses were conducted by Kruskal–Wallis test followed by Dunn’s multiple comparisons test (**B** and **E**) or one-way analysis of variance followed by Dunnett’s multiple comparison test (**C** and **F**).

Our previous study demonstrated an interaction between macrophages and chondrocytes in experimental OA mice. Herein, we evaluated the direct effect of PTX3 on chondrocyte metabolism as well as a possible indirect effect through macrophage modulation. Treatment of mouse primary chondrocytes with rmPTX3 did not induce metabolic disorder. However, treatment of mouse primary chondrocytes with IL-1β increased MMP13 and decreased COL2 and SOX9 (Fig. 4C).

Tibial plateau cartilage explants from 3-week-old mice were cultured in vitro and stimulated with rmPTX3. No metabolic disorder or proteoglycan loss was noted when induced by rmPTX3 (Fig. 4A, B and Supplementary Fig. 4A, B). These results demonstrated that PTX3 had no direct effect on chondrocytes catabolism or anabolism. However, when cartilage explants were treated with supernatants derived from macrophages stimulated with PTX3, significant proteoglycan loss was noted (Fig. 4D, E and Supplementary Fig. 4C, D). Quantitative polymerase chain reaction (qPCR) showed that cultured supernatants of macrophages treated with rmPTX3 promoted the expression of MMP13 and inhibited COL2 and SOX9 in primary chondrocytes (Fig. 4F). These results suggested that PTX3 did not have a direct effect on chondrocytes, but rather, the anti-chondrogenic effects of PTX3 involving OA development were due to macrophages.

### PTX3 promotes OA by interacting with CD32 on macrophages

**Fig. 5:**
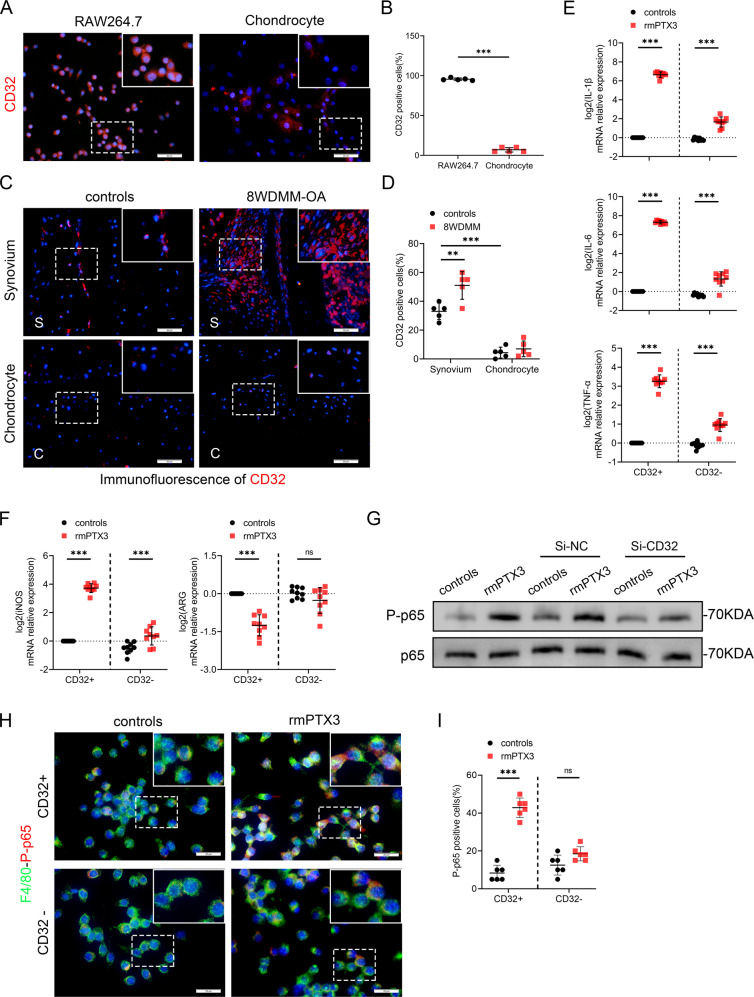
PTX3 promotes OA by interaction with macrophage CD32. **A**, **B** Immunofluorescent staining and quantification of CD32 for RAW264.7 and chondrocytes, *n* = 5 per group. Scale bar: 50 µm. **C**, **D** Immunofluorescent staining and quantification of CD32 of synovial tissues (upper) and knee cartilage (lower) from controls and 8 week-DMM-OA mice, *n* = 5 per group. Scale bar: 50 µm. **E** Relative mRNA expression levels of IL-1β, IL-6 and TNF-α in Si-CD32- or rmPTX3-treated RAW264.7 cells, *n* = 9 per group. **F** Relative mRNA expression levels of iNOS and ARG in Si-CD32- or rmPTX3-treated RAW264.7 cells, *n* = 9 per group. **G** Immunoblotting of P-p65 and p65 of RAW264.7 cells treated with Si-CD32 or rmPTX3. **H**, **I** IF staining and quantification of F4/80 and P-p65 in RAW264.7 treated with Si-CD32 or rmPTX3, *n* = 6 per group. Scale bar: 25 µm. *P < 0.05, **P < 0.01, ***P < 0.001, ns not significant. Data are shown as means ± SD. Statistical analyses were conducted by unpaired *t*-test (**B**) or two-way analysis of variance followed by Sidak’s multiple comparison test (**D**, **E**, **F**, and **I**). Boxed area is enlarged in the top right corner.

It has been reported that PTX3 regulates macrophage physiology and pathology through its surface receptor, CD32 [26–29], although this finding has not been reported for OA. CD32 was demonstrated to be highly expressed by macrophages but not by primary chondrocytes (Fig. 5A, B). CD32-positive cells were abundantly expressed in the synovial tissue of mice 8 weeks post DMM surgery, compared to controls. However, CD32 was poorly expressed in articular cartilage of both OA mice and controls, which may be why PTX3 had no direct effect on chondrocytes (Fig. 5C, D and Supplementary Fig. 5A, B). Further, the effect of PTX3 on M1 macrophages was diminished with CD32 deficiency as was the expression of the pro-inflammatory cytokines IL-1β, IL-6, and TNF-α (Fig. 5E, F and Supplementary Fig. 5C). In contrast, inhibition of another PTX3-related receptor, P-selectin (CD62P) [30, 31], did not affect the regulatory effect of PTX3 on macrophage polarization (Supplementary Fig. 5F, G).

We next evaluated CD32 induced intracellular signal transduction. The NF-κB pathway is a well explored inflammatory pathway that is over activated in OA, exacerbating chondrocyte matrix degradation, synovial inflammation, and macrophage M1 polarization [17, 32–34]. We found macrophage PTX3 stimulation to highly activate p65 NF-κB signaling in macrophages (Supplementary Fig. 5D, E). Silencing of CD32 rescued the phosphorylation of p65 mediated NF-κB signaling (Fig. 5G–I). In summary, PTX3 aggravated OA by interaction with macrophage CD32, activating the p65/NF-κB pathway to promote M1 polarization and cartilage degradation. The paracrine interaction between macrophages and chondrocytes is a feedback loop that enhances synovitis and cartilage damage.

### MiR-224-5p regulates PTX3 during OA development

**Fig. 6:**
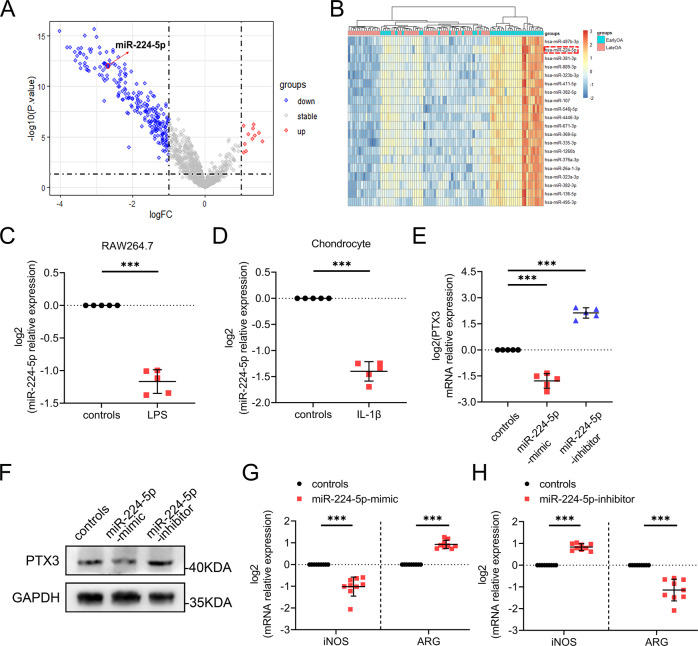
MiR-224-5p regulates PTX3 during OA development. **A** Differentially expressed microRNAs in plasma from early and late knee OA patients based on GSE151341. **B** Twenty downregulated microRNAs from plasma of early and late knee OA patients based on GSE151341. **C** Relative mRNA expression level of miR-224 in RAW264.7 cells treated with LPS, *n* = 5 per group. **D** Relative mRNA expression level of miR-224 in primary murine chondrocytes treated with IL-1β, *n* = 5 per group. **E**, **F** Relative mRNA expression levels and immunoblotting of PTX3 in miR-224-mimic- and miR-224-inhibitor-treated RAW264.7 cells. **G**, **H** Relative mRNA expression levels of iNOS and ARG in RAW264.7 cells treated with LPS, miR-224-mimic, and miR-224-inhibitor, *n* = 9 per group. *P < 0.05, **P < 0.01, ***P < 0.001, ns not significant. Data are shown as means ± SD. Statistical analyses were conducted by unpaired *t*-test (**C**, **D**, **G**, and **H**) or one-way analysis of variance followed by Dunnett’s multiple comparison test (**E**).

Analysis was completed to determine whether microRNA regulates OA by targeting PTX3. Previously, miR-224-5p was found to directly bind PTX3 mRNA [35]. For analysis of miR-224 in OA, we analyzed dataset GSE151341, which is comprised of the plasma miRNome of 91 patients with early and advanced knee OA. Bioinformatic analysis of GSE151341 showed that compared to early OA, miR-224-5p was significantly downregulated in late OA (Fig. 6A, B). QPCR demonstrated expression of miR-224-5p to be significantly downregulated in both LPS-treated macrophages and IL-1β-treated chondrocytes (Fig. 6C, D). Further, macrophage expression of PTX3 was inhibited by a miR-224-5p mimic and promoted by a miR-224-5p inhibitor (Fig. 6E, F). The role of miR-224-5p in macrophage polarization was assessed. The expression of iNOS was downregulated and ARG upregulated with miR-224-5p mimic treatment (Fig. 6G). In contrast, macrophage M1-polarization was enhanced and M2-polarization inhibited with the miR-224-5p inhibitor (Fig. 6H). Taken together, these results demonstrated PTX3 to be negatively regulated by miR-224-5p, which in turn promoted M1 macrophage polarization that exacerbated cartilage damage.

## Discussion

**Fig. 7:**
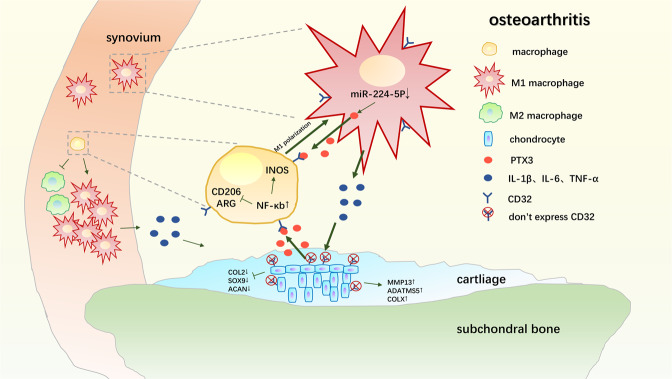
Model of PTX3 regulation of synovial macrophage polarization and disrupted chondrocyte homeostasis during OA. Decreased miR-224-5p promotes M1-polarized OA macrophage and chondrocyte secretion of PTX3. Increased PTX3 promotes increased M1 polarization of synovial macrophages and the secretion of inflammatory cytokines that disrupt chondrocyte homeostasis, accelerating OA progression.

In this study, we established the essential role of PTX3 in aggravating OA occurrence and development. PTX3 had no direct effect on chondrocytes, but did activate the NF-κB pathway in macrophages through CD32, promoting M1 macrophage polarization and the abundant secretion of pro-inflammation cytokines and severe degeneration of articular cartilage. Intra-articular injection of PTX3 aggravated, while PTX3 neutralization reversed, synovitis and cartilage damage. Further, PTX3 expression during OA was demonstrated to result in the downregulation of miR-224-5p (Fig. 7). These findings demonstrate a functional OA developmental pathway and suggest that blockade of PTX3 may be a therapy for OA prevention and treatment.

PTX3 is induced by inflammation and is secreted by inflammatory cells [36]. PTX3 directly participates in inflammation in a variety of ways by inducing the production of pro-inflammatory mediators and by the regulation of complement activation [18]. Further studies confirmed that PTX3 can amplify the complement-mediated mechanism, leading to inflammation and tissue damage [37]. Studies have found that PTX3-positive RA patients with local joint inflammation is more serious than PTX3-negative patients [38]. Kato et al. found that the plasma PTX3 concentration in patients with active IBD was significantly higher than that in inactive IBD patients and healthy people [39]. In SLE, PTX3 can accelerate vascular injury through TNF- α / NF- κ b signal pathway [40]. In addition, the deposition degree of PTX3 in lupus nephritis (LN) is also related to the pathological degree [41]. Inhibition of PTX3 expression can slow down the abnormal proliferation of mesangial cells on LN and effectively reduce renal damage [42]. Accumulating evidence suggests that synovial inflammation is related to the pathogenesis and progression of OA and has a significant correlation with joint pain [43–45], but a relationship between PTX3 and OA has not been reported previously. In this study, PTX3 was found to be highly expressed in the synovium and chondrocytes of patients with OA as well as in an experimental mouse model of OA. We also found that intra-articular injection of rmPTX3 aggravated synovitis and cartilage destruction in the DMM-OA mouse model. Administration of neutralizing PTX3 antibody effectively reversed synovitis and partially protected from cartilage destruction. These results suggest that PTX3 is an important contributor to OA occurrence and development.

Emerging evidence has shown that an imbalance of M1/M2 macrophage polarization is essential to the inflammation associated with OA [46]. Our previous study showed that M1 but not M2-polarized macrophages accumulated in human and mouse OA synovial tissue [17]. Using a transgenic mouse model with enhanced M1- or M2-polarized macrophages, we found synovial macrophage M1 polarization to exacerbate experimental collagenase-induced OA, while M2 polarization attenuated OA. The findings identified a critical role for synovial M1 and M2 macrophages in the development of OA. PTX3 has been reported to modulate macrophage polarization [5, 10]. This study found that exogenous PTX3 reduced M2 macrophage polarization and promoted M1 polarization, increasing the release of inflammatory cytokines during the development of OA. Macrophage polarization impacted the metabolic balance of chondrocytes and eventually aggravated OA. When tibial plateau explants were treated with rmPTX3, there was no effect on chondrocyte synthesis or metabolism of cartilage. However, co-culture with supernatants of macrophages stimulated with PTX3, chondrocyte metabolism was significantly affected, suggesting that PTX3 aggravates OA by the reprogramming of macrophages.

Recent studies have demonstrated a strong link between the NF-κB pathway and enhanced M1 macrophage polarization [47]. Further, during OA inflammation, a large number of damage-associated molecular patterns (DAMPs) and pathogen-associated molecular pattern (PAMPs) are released, which activate macrophages to produce pro-inflammatory cytokines such as IL-1β, IL-6 and TNF-α through the NF-κB pathway [48–51]. Herein, we found NF-κB signaling to be involved in an OA associated phenotype, activated by PTX3. PTX3 can interact with the Fcγ receptor (FCγRII/CD32) on cell surfaces to activate physiological and pathological functions such as leukocyte-mediated phagocytosis [28, 52]. CD32, i.e. FCγRII, has various forms with differing function. FCγRII A (CD32A) induces cell excitation through intracellular signal transduction, while FCγRII B (CD32B) transduces inhibitory signals [28, 53]. This study found CD32 to be highly expressed by macrophages, but poorly expressed by chondrocytes, which likely explains why PTX3 had no direct effect on chondrocytes. When Mus-siRNA was used to knock down CD32B in RAW264.7 cells, we accidentally found that RAW264.7 cells also expressed CD32A, and like CD32B, could be successfully inhibited by Mus-siRNA (Supplementary Fig. 5C). This study is the first to detect CD32A expression in mice. Further, this study clarified and explained the OA effects of the NF-κB signal pathway, demonstrating that PTX3 activates the P-p65/NF-κB pathway through CD32, which mediates macrophage polarization and inflammation. In contrast, P-selectin, another receptor of PTX3 [30, 31], does not play a key role in PTX3-mediated macrophage reprogramming.

We also found that an increase in PTX3 was related to the development of OA. We explored the reasons for this by analysis of microRNA. Many studies have demonstrated microRNA to be involved in the occurrence and development of OA [54, 55]. We speculated that the increase in OA PTX3 was related to an abnormal decrease in some microRNA. In previous studies, miR-224-5p was found to target PTX3 [56]. We found expression of miR-224-5p to be downregulated in late OA and to be differentially expressed in early and late OA patients. Other studies have reported that miR-224-5p is involved in the occurrence and development of other diseases. For example, in cervical cancer, inhibition of miR-224-5p prevents disease progression by targeting PTX3 [35]. In this study, we found that miR-224-5p was significantly downregulated in chondrocytes stimulated with IL-1β and as well M1-polarized macrophages induced by LPS. We also found that miR-224-5p regulates polarization and PTX3 expression in RAW264.7 cells, suggesting that miR-224-5p may play a role in macrophage polarization and OA by targeting PTX3.

In summary, we found downregulation of miR-224-5p in OA leads to increased expression of PTX3, which mediates synovial macrophage polarization and inflammation through CD32, ultimately aggravating OA. Analysis of the OA cyclical interaction of chondrocytes, PTX3, and macrophages will provide for a novel approach by which to investigate the development of OA. Although the effect of inhibiting PTX3 on OA has not been clinically confirmed, control of macrophage polarization and inflammatory secretion may be an effective strategy by which to treat OA.

## Materials and methods

### Human sample

The study was approved by the Ethics Committee of the third affiliated Hospital of Southern Medical University, and on the premise of informed consent. The sample was drilled from the medial and lateral tibial plateau of five patients with osteoarthritis who underwent total knee arthroplasty, in which the medial tibial plateau was the OA group and the lateral tibial plateau cartilage was less damaged as the NC group. Normal synovium was taken from 5 road traffic accident victims with no history of arthritis, OA synovium was taken from 5 patients undergoing total knee arthroplasty, and preoperative serum and synovial fluid of the above patients were collected as control group or OA group respectively. All the above patients were treated in the third affiliated Hospital of Southern Medical University, and the collection of samples was completed by the same person.

### Animals

All animal were purchased from the Experimental Animal Centre of Southern Medical University (Guangzhou, China). All animal experiments were approved by the Southern Medical University Committee Animal Care and Use Committee, and were performed in accordance with the Committee’s regulations.

### OA model

All animals were randomly assigned to each group before the experiment. Ten-week-old male C57/BL6 mice (*n* = 5) were subjected to destabilization of the medial meniscus (DMM) surgery to induce OA. Five days after surgery all DMM-OA mice were divided into three groups(*n* = 5): the treatment group was injected with recombinant mouse PTX3 (Cloud-Clone, Wuhan, China) 5ug/5uL/week into the right knee joint cavity (DMM + rmPTX3), the vehicle group was injected with PTX3 neutralizing antibody (Abbkine, Wuhan, China) 5ug/5uL/week into the right knee joint cavity (DMM + PTX3-NAb), and the control group was injected with the same amount of normal saline every week (DMM). At postoperative weeks 4 and 8, mice were killed for collection of knee joint specimens.

### Cell

Primary murine chondrocytes were derived from tibial plateaus of 7-day-old C57 mice, and be cultured in Dulbecco’s modified Eagle’s medium F12 (DMEM: F12) (Gibco, Carlsbad, CA, USA) with 20% fetal bovine serum (Gibco) and 1% Penicillin-Streptomycin at 37 °C under 5% CO_2_. Mouse macrophage-like RAW264.7 cell from the American Type Culture Collection (ATCC, Manassas, VA, USA) were grown in Dulbecco’s modified Eagle’s medium (DMEM) with 4.5 g/L glucose (Gibco) with 10% fetal bovine serum. Primary murine chondrocytes were treated with 10 ng/mL interleukin-1β (IL-1β) (R&D systems, Minneapolis, MN, USA) and 500 ng/mL lipopolysaccharide (LPS) (Invitrogen, San Diego, CA, USA) for 48 h to create the model of OA chondrocytes in vitro. RAW264.7 cells were treated with 500 ng/mL LPS for 24 h to induce M1 polarization. RAW264.7 cells were treated with 50 ng/mL IL-4 (R&D systems) for 24 h to induce M2 polarization. To primary chondrocytes were treated with 200 ng/mL or 400 ng/mL rmPTX3 and RAW264.7 cells were treated with 2 ng/mL, 20 ng/mL, 200 ng/mL or 400 ng/mL rmPTX3.

### Cartilage explants

Tibial plateaus cartilage explants were isolated from 3-week-old male C57 mice. Use eye scissors to cut off the tibial plateau at the position of the growth plate below the tibial plateau and remove soft tissues such as synovium under a microscope. Explants were cultured in DMEM/F12 medium containing 20% fetal bovine serum in 48-well plate for 3 days. After 3 days, the activity of the explant was judged according to the color change of the culture medium, and then treated the explants with 10 ng/ml IL-1 β, 200 ng/ml rmPTX3 and 400 ng/mL rmPTX3 respectively for 3 days.

### Co-culture

Raw264.7 cells were stimulated with IL-1 β of 10 ng/ml, rmPTX3 of 200 ng/ml and rmPTX3 of 400 ng/mL for 24 h, and the supernatant was collected and co-cultured with primary chondrocytes in 12-well plate (500 μl supernatant and 500 μl DMEM/F12 containing 20% fetal bovine serum) for 48 h. In addition, the supernatant was co-cultured with tibial plateau explants in 48-well plate (250 μL supernatant and 250 μL DMEM/F12 containing 20% fetal bovine serum) for 3 days.

### Histological analysis

The knee joint was fixed with 4% paraformaldehyde for 24 h and 0.5 M EDTA was decalcified at 37 °C and pH7.4 for 14 days and embedded in paraffin. Three-micrometer-thick sections were cut for both hematoxylin and eosin (H&E) and Safranin O/Fast Green (Saf-O) staining. Synovitis severity was estimated by two blinded observers based on the density of the resident cells [1–3], inflammatory infiltration [1–3] and enlargement of the synovial lining cell layer [1–3] and the sum of the three scores is presented (maximum site score 9) [57]. Safranin O/Fast Green stain sections were assigned a grade of 0–6 by two blinded observers to grade cartilage degeneration based on the Osteoarthritis Research Society International (OARSI) scoring system developed [58]. Safranin O/Fast Green of cartilage explants were graded by two blinded observers based on the area of staining loss by using a 6-point scale modified from previously published systems: grade 0 (none loss), grades 1–3 (Loss of staining affecting ≤1/2 of the cartilage depth of explants and involving ≤1/3, >1/3 but ≤2/3, or >2/3 of the plateau, respectively), grades 4–6 (Loss of staining affecting >1/2 of the cartilage depth of explants and involving ≤1/3, >1/3 but ≤2/3, or >2/3 of the plateau, respectively) [59].

### Immunohistochemistry (IHC)/immunofluorescence (IF) staining

Specimens were prepared as described above and sections were soaked in citrate buffer (10 mM citric acid, pH 6.0) overnight at 60 °C to unmask antigen.

For immunohistochemical staining (IHC), 3% hydrogen peroxide were added for 10 min to inactivate endogenous peroxidase activity. Sections were blocked with 1% goat serum (Solarbio, Beijing, China) at 37 °C for 1 h and incubated with primary antibodies at 4 °C for 12 h. Then the sections were incubated with species-matched horseradish peroxidase-conjugated secondary antibodies (J Jackson ImmunoResearch Laboratories, West Grove, PA, USA) at room temperature for 1 h, then 3, 3-diaminobenzidine (DAB) was used to observe the chromogen, with hematoxylin for counterstaining.

For immunofluorescence staining (IF), secondary antibodies conjugated with Alexa Fluor 488 or Alexa Fluor 594 (Life Technologies, Carlsbad, CA, USA) were added and incubated at room temperature for 1 h in dark and the nuclei were labeled with 4, 6-diamidino-2-phenylindole (DAPI; Thermo Fisher Scientific, Waltham, MA, USA).

The number of positively stained cells in two fields per section and three sequential sections per mouse were counted in each group. The following primary antibodies were used: rabbit anti-PTX3 (1:150 for IHC, 1:200 for IF; Proteintech, Wuhan, China, 13797-1-AP), rabbit anti-COLX (1:100 for IHC; Abcam, Cambridge, UK, ab58632), rabbit anti-SOX9 (1:200 for IHC; Abclonal, Woburn, MA, USA, A19170), rabbit anti-ACAN (1:200 for IHC; Abclonal, A11691), rabbit anti-ADATMS5 (1:300 for IF; Affinity Biosciences, USA, DF13268), rabbit anti-MMP13 (1:400 for IF; Affinity Biosciences, AF5355), rabbit Anti-COL2 (1:200 for IF; Abcam, ab34712), rabbit anti-CD206 (1:100 for IF; Proteintech, 18704-1-AP), rabbit anti-iNOS (1:100 for IF; Santa Cruz, Texas, USA, sc-7271), mouse anti-F4/80 (1:100 for IF; Santa Cruz, sc-377009), mouse anti-CD32 (1:200 for IF; BD Pharmingen™, USA, 553142)

### Cell IF staining

RAW264.7 cells cultured at 20 mm round coverslip were treated with 400 ng/mL rmPTX3 for 12 h and 24 h. RAW264.7 cell and primary murine chondrocytes cultured at 24 mm round coverslip with no treatment. Treated the cells with 4% paraformaldehyde for 15 min, then blocked the cells with 1% sheep serum at 37 °C for 30 min and incubated with primary antibodies (in 1% BSA, 0.1% Triton X-100) at 4 °C for 12 h. Then the cells were incubated with secondary antibodies conjugated with Alexa Fluor 488 or Alexa Fluor 594 at room temperature for 1 h in dark and the nuclei were labeled with 4, 6-diamidino-2-phenylindole. The number of positively stained cells of total cell count per round coverslip were counted in each group. The following primary antibodies were used: rabbit anti-phosphorylated p65 and P-p65 (1:100 for cell IF; CST, Danvers, MA, USA, 8242 and 3033), mouse anti-F4/80 (1:100 for cell IF; Santa Cruz, sc-377009), mouse anti-CD32 (1:200 for cell IF; BD Pharmingen™, USA, 553142)

### Quantitative reverse transcription-polymerase chain reaction (RT-qPCR)

Total RNA was extracted from primary murine chondrocytes, RAW264.7 cells using TRIzol reagent (Takara Bio Inc., Shiga, Japan). cDNA was reverse transcribed using 5× HiScript II qRT Super-Mix II (Vazyme Biotech, Nanjing, China) and PCR was performed using 10 µL 2× ChamQ SYBR qPCR Master Mix (Vazyme) on a light cycler (Roche, Basel, Switzerland) with the following primers: mouse GAPDH (forward primer 5′-AGG TCG GTG TGA ACG GA T TTG-3′, reverse primer 5′-TGT AGA CCA TGT AGT TGA GGT CA-3′), mouse iNOS (forward primer 5′-GTT CTC AGC CCA ACA ATA CAA GA-3′, reverse primer 5′-GTG GAC GGG TCG ATG TCA C-3′), mouse Arg (forward primer 5′-TTG GGT GGA TGC TCA CAC TG-3′, reverse primer 5′-GTA CAC GAT GTC TTT GGC AGA-3′), mouse IL-1β (forward 5′-GCA ACT GTT CCT GAA CTC AAC T-3′, reverse 5′-ATC TTT TGG GGT CCG TCA ACT-3′), mouse IL-6 (forward 5′-ACA ACC ACG GCC TTC CCT ACT T-3′, reverse 5′-CAG GAT TTC CCA GCG AAC ATG TG-3′), mouse TNF-α (forward 5′-CCT CCC TCT CAT CAG TTC TA-3′, reverse 5′-ACT TGG TTT GCT ACG AC-3′), mouse MMP13 (forward primer 5′-CTT CTT GTT GAG CTG GA CTC-3′, reverse primer 5′-CTG TGG AGG TCA CTG TAG ACT-3′), mouse COL2 (forward primer 5′-CAC CCT CAA ATC CCT CAA CAA TCA G-3′, reverse primer 5′-TGT CTT TCG TCT TGC TGG TCC ACC-3′), mouse SOX9 (forward primer 5′-TAC CTA CGG CAT CAG CAG CTC-3′, reverse primer 5′-TTG CCT TCA CGT GGC TTT AAG-3′), mmu-miR-224-5p (forward primer 5′-TCC GCT AAG TCA CTA GTG GTT C-3′, reverse primer 5′-CAG AGC AGG GTC CGA GGT A-3′), mouse CD62P (forward primer 5′-CAT CTG GTT CAG TGC TTT GAT CT-3′, reverse primer 5′-ACC CGT GAG TTA TTC CAT GAG T-3′).

### Enzyme-linked immunosorbent assay (ELISA)

We used the Mouse IL-1β, IL-6 and TNF-α ELISA Kits (Elabscience Biotechnology, Bethesda, MD, USA, E-EL-M0037c, E-EL-M0044c and E-EL-M0049c) to analyze IL-1β, IL-6 and TNF-α levels in the supernatants of macrophage treated with 400 ng/mL rmPTX3. Human PTX3 ELISA Kit (Elabscience Biotechnology, E-EL-H6081) was used to analyze PTX3 levels in the preoperative serum and synovial fluid of patients. Mouse PTX3 ELISA Kit (Animalunion Biotechnology, Shanghai, China, LV30443) was used to analyze PTX3 levels in the preoperative serum of control and OA mice.

### Western blot analysis (WB)

RAW264.7 cells cultured in 12-well dishes were lysed with 100 μL of radioimmunoprecipitation assay (RIPA) buffer (Beyo-time Institute of Biotechnology, Jiangsu, China) containing protease inhibitor and phosphatase inhibitor. Cell lysates were analyzed by sodium dodecyl sulfate-polyacrylamide gel electrophoresis and transferred to a nitrocellulose membrane (Bio-Rad Corp, Hercules, CA, USA). Blots were probed with primary antibodies and immunoreactive proteins were revealed using an enhanced chemiluminescence kit (Santa Cruz). The following primary antibodies for WB were used: rabbit anti-PTX3 (1:1000 for WB; Proteintech, 13797-1-AP), rabbit anti-GAPDH (1:10,000 for WB; Abcam, ab181603), rabbit anti-FCGR2A/CD32A (1:1000 for WB; Proteintech, 15625-1-AP), rabbit anti-FCGR2B/CD32B (1:1000 for WB; Affinity, DF7666), rabbit anti-P-p65 (1:1000 for WB; CST, 3033), rabbit anti-p65 (1:1000 for WB, CST, 8242).

### SiRNA transfection

Inhibition of CD32 mRNA was achieved with the use of small interference RNA (siRNA). 75 nanomolar CD32 siRNA or siRNA negative control (Si-NC) (GenePharma, Suzhou, China) were transfected into RAW264.7 cells using lipofectamine 3000 (3 μL/mL) for 24 h following the manufacturer’s protocols, then treated the RAW264.7 cells with 400 ng/mL rmPTX3 for 24 h (qPCR and WB) or 6 h (cell IF). Then the cells were processed with Trizol for RNA analysis, RIPA for western blot analysis or 4% paraformaldehyde for cell IF as described above.

Si-CD32-183 (forward: 5′-GGG ACU CAU GAU CUU CCA ATT-3′, reverse: 5′-UUG GAA GAU CAU GAG UCC CTT-3′);

Si-CD32-762 (forward: 5′-GCU GUC GCA GCC AUU GUU ATT -3′, reverse: 5′-UAA CAA UGG CUG CGA CAG CTT-3′);

Si-CD32-948 (forward: 5′-CCA ACA AGC AGC AGC CCA UTT -3′, reverse: 5′-AUG GGC UGC UGC UUG UUG GTT-3′);

Si-NC (forward: 5′-UUC UCC GAA CGU GUC ACG UTT -3′, reverse: 5′-ACG UGA CAC GUU CGG AGA ATT-3′).

Inhibition of CD62P (P-selectin) mRNA was achieved with the use of small interference RNA (siRNA). 75 nanomolar CD62P siRNA or siRNA negative control (Si-NC) (Tsingke Biotechnology, Beijing, China) were transfected into RAW264.7 cells using lipofectamine 3000 (3 μL/mL) for 24 h following the manufacturer’s protocols, then treated the RAW264.7 cells with 400 ng/mL rmPTX3 for 24 h (qPCR). Then the cells were processed with Trizol for RNA analysis as described above.

Si-CD62P-1 (forward: 5′- CCA UGU GCA GAG CGG UCA ATT -3′, reverse: 5′- UUG ACC GCU CUG CAC AUG GTT -3′);

Si-CD62P-2 (forward: 5′- GCG UGG ACC UAU AAC UAC ATT -3′, reverse: 5′- UGU AGU UAU AGG UCC ACG CTT -3′);

Si-CD62P-3 (forward: 5′- CCG AAC ACC ACU UGU UAC UTT -3′, reverse: 5′- AGU AAC AAG UGG UGU UCG GTT -3′);

Si-CD62P-4 (forward: 5′- CAG AGU GUG AAU ACG UCA ATT -3′, reverse: 5′- UUG ACG UAU UCA CAC UCU GTT -3′);

Si-NC (forward: 5ʹ-UUC UCC GAA CGU GUC ACG UTT -3′, reverse: 5′-ACG UGA CAC GUU CGG AGA ATT-3′).

### miR-224-5p mimics and inhibitor transfection

50 nanomolar miR-224-5p mimics or miR-NC (controls) (TsingKe, Beijing, China) were transfected into RAW264.7 cells treated with 500 ng/mL LPS using lipofectamine 3000 (3 μL/mL) for 24 h following the manufacturer’s protocols. 80 nanomolar miR-224-5p inhibitor or miR-NC (TsingKe, Beijing, China) were transfected with Lipofectamine 3000 into RAW264.7 cells treated with 500 ng/mL LPS. Then the cells were processed with Trizol for RNA analysis or RIPA for western blot analysis as described above.

miR-224-5p mimics (forward: 5′-UAA GUC ACU AGU GGU UCC GUU-3′, reverse: 5′-AAC GGA ACC ACU AGU GAC UUA-3′);

miR-224-5p inhibitor (5′-AAC GGA ACC ACU AGU GAC UUA-3′);

miR-NC (forward:5′-UUC UUC GAA CGU GUC ACG UTT-3′, reverse:5′-ACG UGA CAC GUU CGG AGA ATT-3′).

### Bioinformatic analysis

GSE151341 [60], which sequenced and analyzed the plasma miRNome from 91 patients with early [Kellgren–Lawrence grade 0 or 1 (*n* = 41)] or late [Kellgren–Lawrence grade 3 or 4 (*n* = 50)] symptomatic radiographic knee osteoarthritis, was downloaded from the GEO platform. Data were normalized and transformed into regularized log (rlog) format. The Limma package was used to analyze the differentially expressed microRNAs with the log fold change cutoff set as 1.5. The ggplot2 package and pheatmap package were used to draw a volcano plot and heatmap.

### Statistical analyses

All experiments were performed in duplicate or triplicate and observed by independent observers. Adobe Photoshop 2020 was used to process pictures, GraphPad Prism 8.0.2 was used for statistical analysis and graphs generating. The specific statistical analysis methods of all results have been illustrated in the figure legends. *P*-values < 0.05 were considered significant.

## Supplementary information

Supplementary Figure Legends

Supplementary Figure 1

Supplementary Figure 2

Supplementary Figure 3

Supplementary Figure 4

Supplementary Figure 5

Original Western Blots Legends

Original Western Blots

Original Western Blots Legends

Original Western Blots

Agreement from all authors about the author list

Reproducibility checklist

## Data Availability

Supplementary Figs. 1–5. and Supplementary Figure Legends have been included in the Supplemental Material 1. Original western blots (Supplementary Fig. 6) have been included in the Supplemental Material 2.
