# Supplementary material for: Pentraxin 3 regulated by miR-224-5p modulates macrophage reprogramming and exacerbates osteoarthritis associated synovitis by targeting CD32
Source: Cell Death Dis. 2022 Jun 24;13(6):567. doi: 10.1038/s41419-022-04962-y (PMC9226026; doi:10.1038/s41419-022-04962-y)
Supplement: Supplementary file 1 — Supplementary Figure Legends [file 41419_2022_4962_MOESM1_ESM.docx]

**Pentraxin 3** **regulated by miR-224-5p modulates macrophage reprogramming and exacerbates osteoarthritis associated synovitis** **by targeting CD32**

**Supplementary Figure Legends**

**Supplementary Figure 1.**

**(A, B)** ELISA of PTX3 in serum and synovial fluid of patients, n=5 per group. **(C)** ELISA of PTX3 in serum of 8W-DMM-OA mice, n=5 per group. **(D)** Immunoblotting of PTX3 from RAW264.7 cells treated with lipopolysaccharide (LPS). **(E)** Immunoblotting of PTX3 from primary murine chondrocytes treated with LPS or IL-1β. *P < 0.05, **P < 0.01, ***P < 0.001, ns not significant. Data are shown as means ± SD. Statistical analyses were conducted by unpaired t test.

**Supplementary Figure 2.**

**(A, B)** IHC staining and quantification of SOX9 in knee cartilage from DMM mice treated with vehicle, rmPTX3, or PTX3-NAb for 8 weeks, n=5 per group. Scale bar: 50 µm. **(C, D)** IHC staining of COLX and ACAN in knee cartilage from DMM mice treated with vehicle, rmPTX3, or PTX3-NAb for 8 weeks, n=5 per group. Scale bar: 50 µm. *P < 0.05, **P < 0.01, ***P < 0.001, ns not significant. Data are shown as means ± SD. Statistical analyses were conducted by one-way analysis of variance followed by Dunnett's multiple comparison test. Boxed area is enlarged in the top right corner.

**Supplementary Figure 3.**

**(A)** Relative mRNA expression level of iNOS in rmPTX3-treated RAW264.7 cells, n=6 per group. **(B)** Relative mRNA expression level of ARG in rmPTX3-treated RAW264.7 cells, n=6 per group. *P < 0.05, **P < 0.01, ***P < 0.001, ns not significant.

**Supplementary Figure 4.**

**(A)** Immunofluorescent staining of MMP13 and COL2 in mouse tibial plateau cartilage explants treated with IL-1β or rmPTX3. Scale bar: 50 µm. **(B)** Quantification of MMP13 in mouse tibial plateau cartilage explants described in (A), n=3 per group. *P < 0.05, **P < 0.01, ***P < 0.001, ns not significant. **(C)** IHC staining of MMP13 and COL2 in mouse tibial plateau cartilage explants co-cultured with supernatants of RAW264.7 (RAW264.7 treated with IL-1β or rmPTX3). Scale bar: 50 µm. **(D)** Quantification of MMP13 in mouse tibial plateau cartilage explants described in **C**, n=3 per group. *P < 0.05, **P < 0.01, ***P < 0.001, ns not significant. Data are shown as means ± SD. Statistical analyses were conducted using one-way analysis of variance followed by Dunnett's multiple comparison test. Boxed area is enlarged in the top right corner.

**Supplementary Figure 5.**

**(A, B)** IHC staining and quantification of CD32 in knee cartilage and synovial tissues from controls and 8 week-DMM-OA mice. Scale bar: 50 µm, 200 µm. **(C)** Immunoblotting of CD32A and CD32B of RAW264.7 cells treated with Si-CD32 (si-CD32-1, 2, 3). **(D, E)** IHC staining and quantification of F4/80 and P-p65 of RAW264.7 cells treated with rmPTX3, n=6 per group. Scale bar: 25 µm. **(F)** Relative mRNA expression level of CD62P of RAW264.7 cells treated with Si-CD62P (si-CD62P -1, 2, 3, 4). **(G)** Relative mRNA expression level of CD62P, iNOS and ARG of RAW264.7 cells treated with Si-CD62P-4 or rmPTX3, n=6 per group. *P < 0.05, **P < 0.01, ***P < 0.001, ns not significant. Data are shown as means ± SD. Statistical analyses were conducted using one-way analysis of variance followed by Tukey's multiple comparison test (B and F) or Dunnett's multiple comparison test (E) or two-way analysis of variance followed by Sidak's multiple comparison test (G). Boxed area is enlarged in the top right corner.
