# Supplementary figures and images for: Pentraxin 3 regulated by miR-224-5p modulates macrophage reprogramming and exacerbates osteoarthritis associated synovitis by targeting CD32
Source: Cell Death Dis. 2022 Jun 24;13(6):567. doi: 10.1038/s41419-022-04962-y (PMC9226026; doi:10.1038/s41419-022-04962-y)

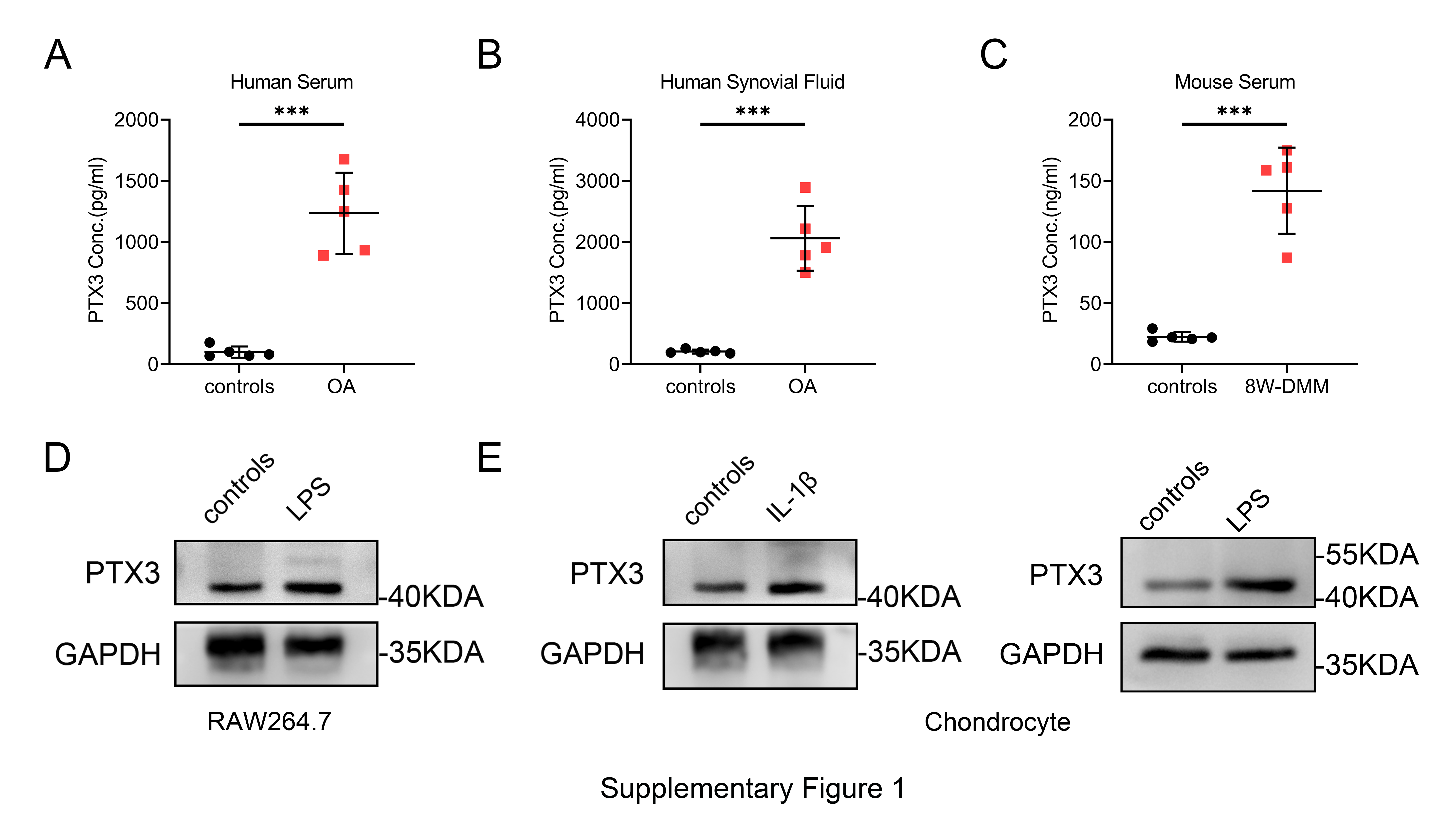

Supplement: Supplementary file 2 — Supplementary Figure 1 [file 41419_2022_4962_MOESM2_ESM.png]

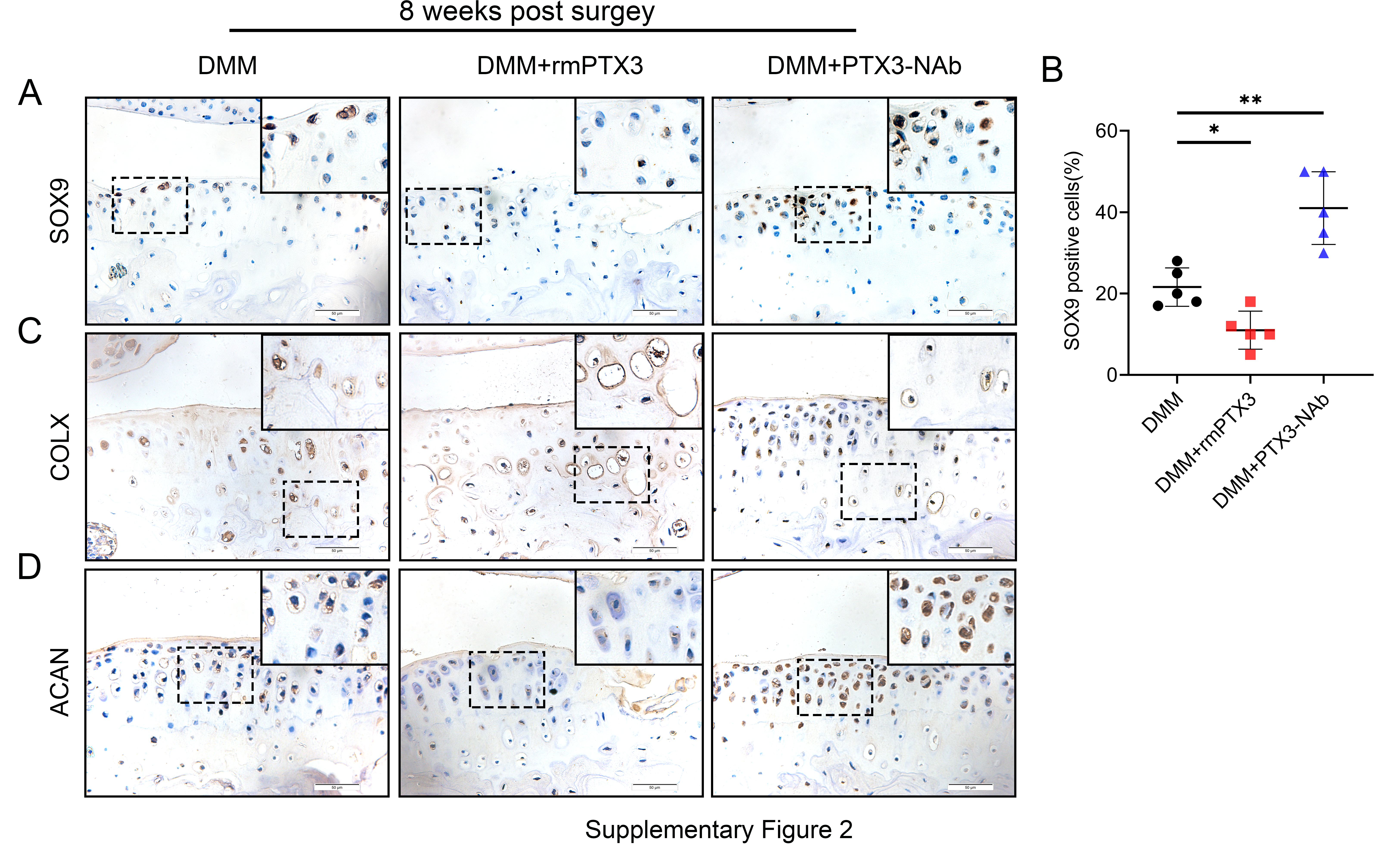

Supplement: Supplementary file 3 — Supplementary Figure 2 [file 41419_2022_4962_MOESM3_ESM.png]

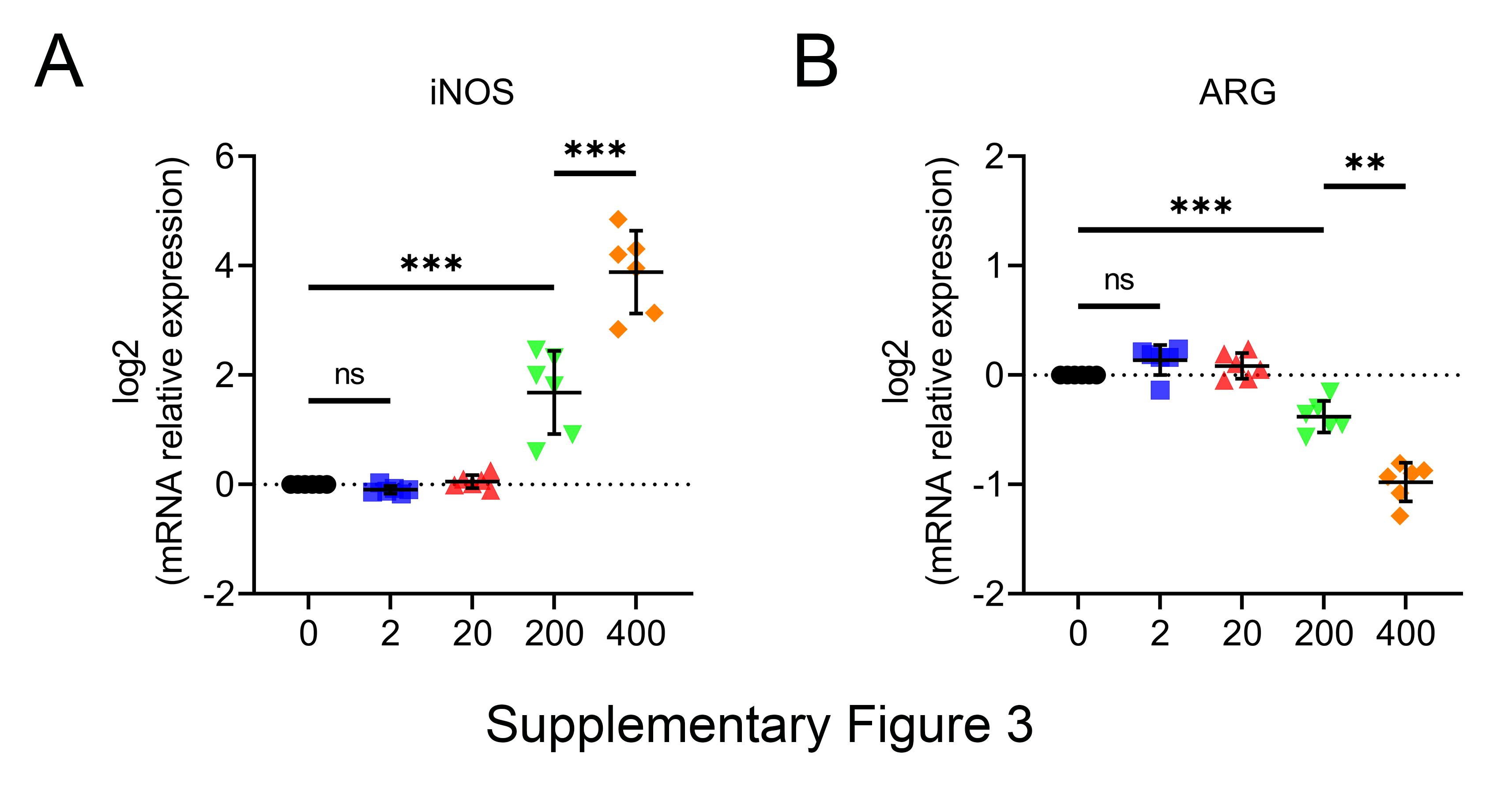

Supplement: Supplementary file 4 — Supplementary Figure 3 [file 41419_2022_4962_MOESM4_ESM.png]

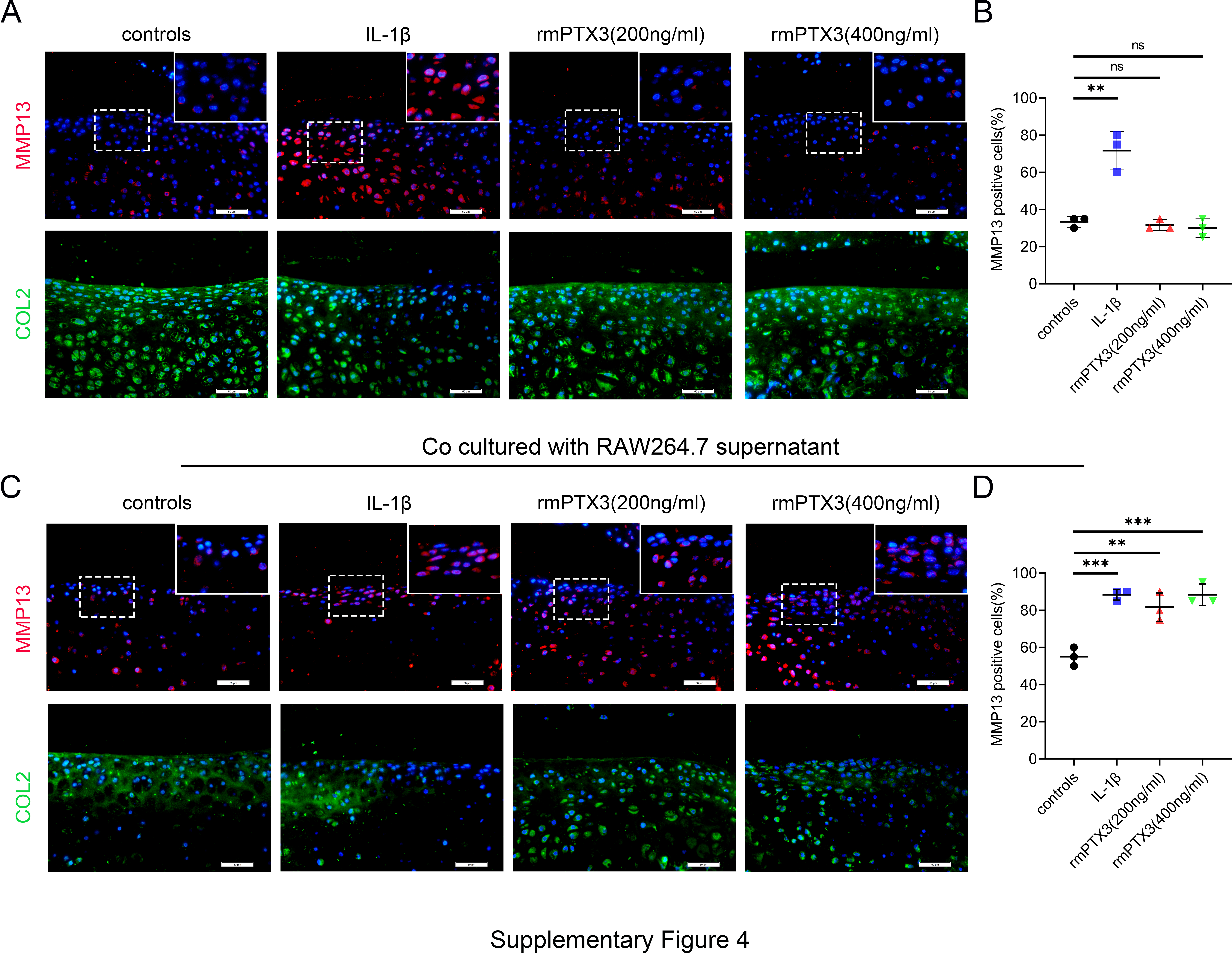

Supplement: Supplementary file 5 — Supplementary Figure 4 [file 41419_2022_4962_MOESM5_ESM.png]

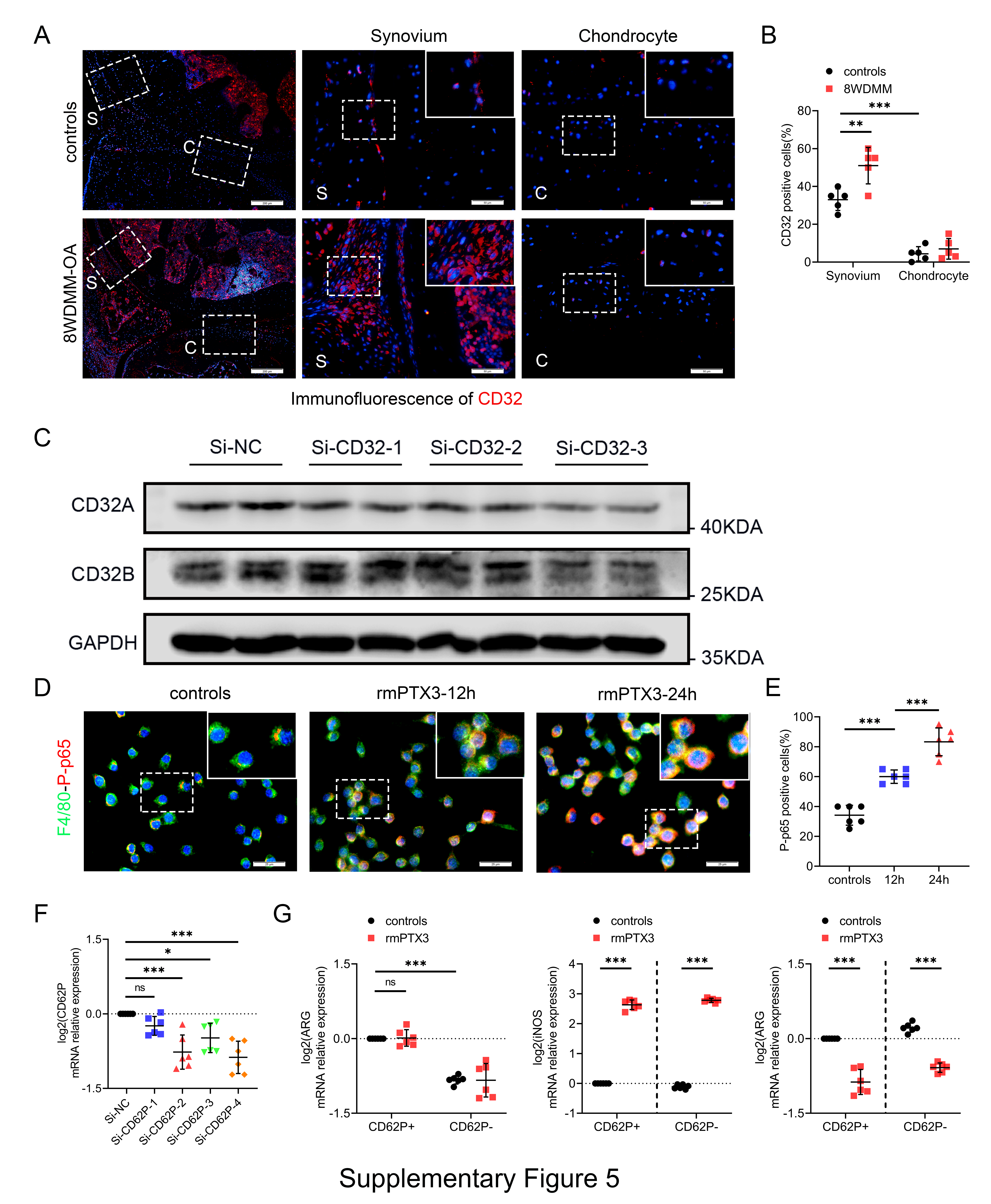

Supplement: Supplementary file 6 — Supplementary Figure 5 [file 41419_2022_4962_MOESM6_ESM.png]

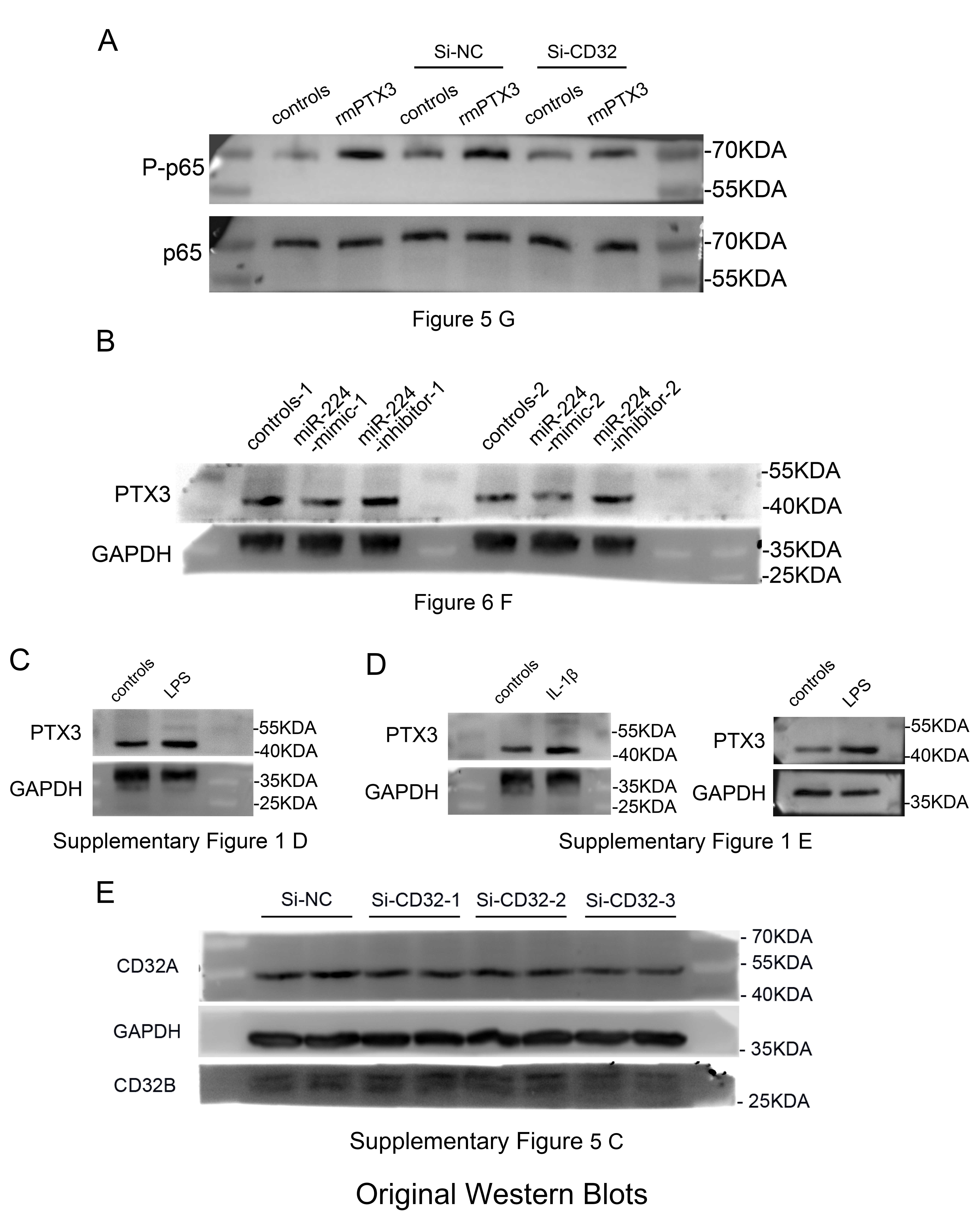

Supplement: Supplementary file 8 — Original Western Blots [file 41419_2022_4962_MOESM8_ESM.png]
