# Supplementary material for: Pentraxin 3 regulated by miR-224-5p modulates macrophage reprogramming and exacerbates osteoarthritis associated synovitis by targeting CD32
Source: Cell Death Dis. 2022 Jun 24;13(6):567. doi: 10.1038/s41419-022-04962-y (PMC9226026; doi:10.1038/s41419-022-04962-y)
Supplement: Supplementary file 7 — Original Western Blots Legends [file 41419_2022_4962_MOESM7_ESM.docx]

**Pentraxin 3** **regulated by miR-224-5p modulates macrophage reprogramming and exacerbates osteoarthritis associated synovitis** **by targeting CD32**

**Original Western Blots Legends**

**Original Western Blots**

**(A)** Original western blots of (Figure 5 G). **(B)** Original western blots of (Figure 6 F). **(C, D)** Original western blots of (Supplementary Figure 1 D, E). **(E)** Original western blots of (Supplementary Figure 5 C).
