# Supplementary material for: Pentraxin 3 regulated by miR-224-5p modulates macrophage reprogramming and exacerbates osteoarthritis associated synovitis by targeting CD32
Source: Cell Death Dis. 2022 Jun 24;13(6):567. doi: 10.1038/s41419-022-04962-y (PMC9226026; doi:10.1038/s41419-022-04962-y)

Mr. Jianbin Yin,

[yinjianbin1996@163.com](mailto:yinjianbin1996@163.com)


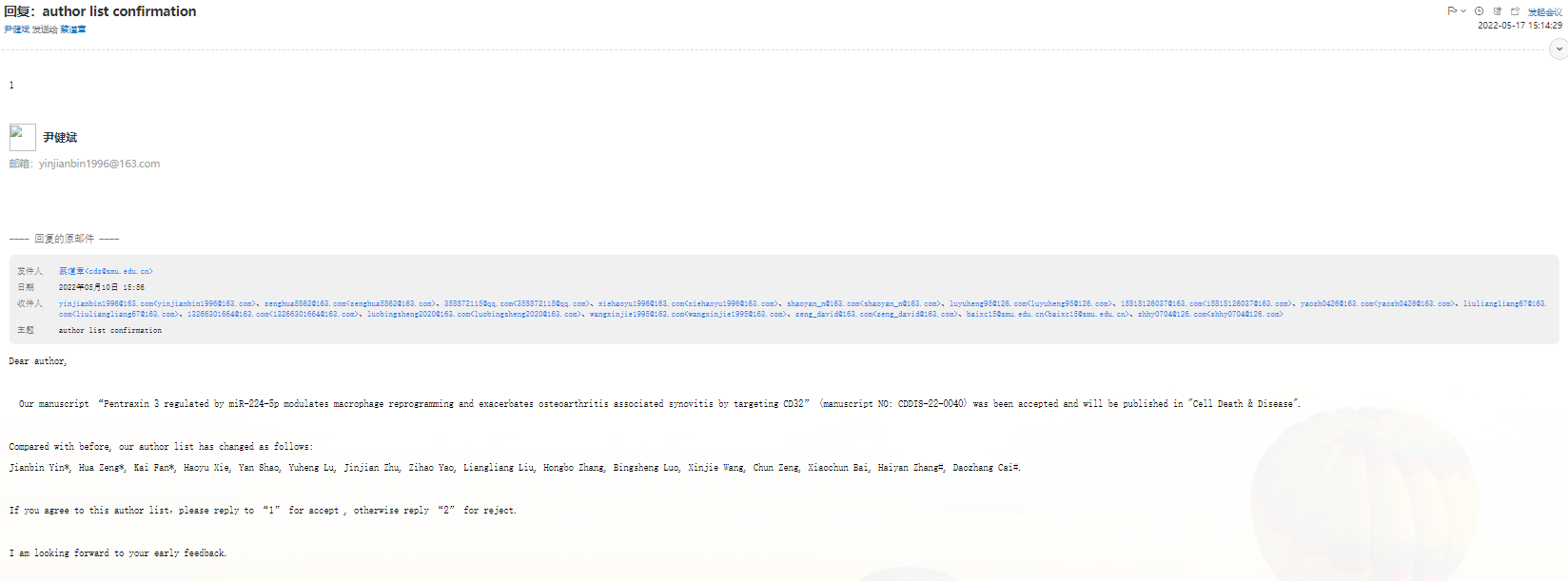


Dr. Hua Zeng

[zenghua5862@163.com](mailto:zenghua5862@163.com)


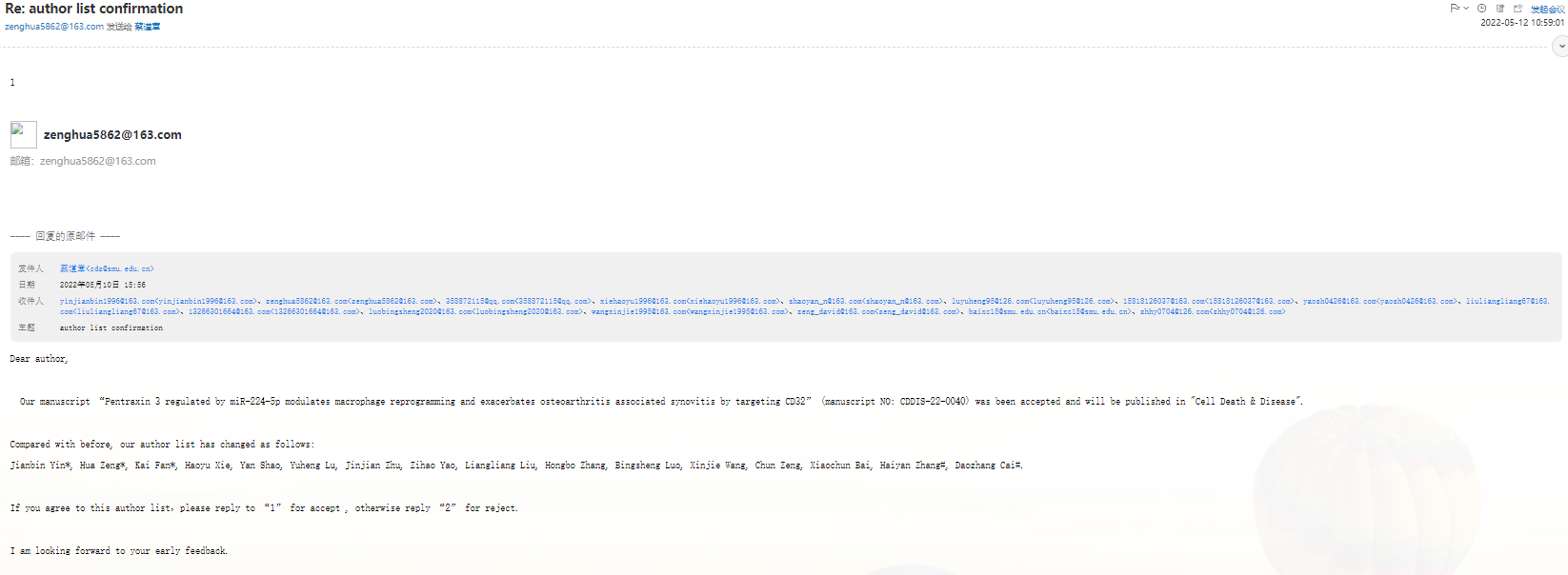


Mr. Kai Fan

[358872115@qq.com](mailto:358872115@qq.com)


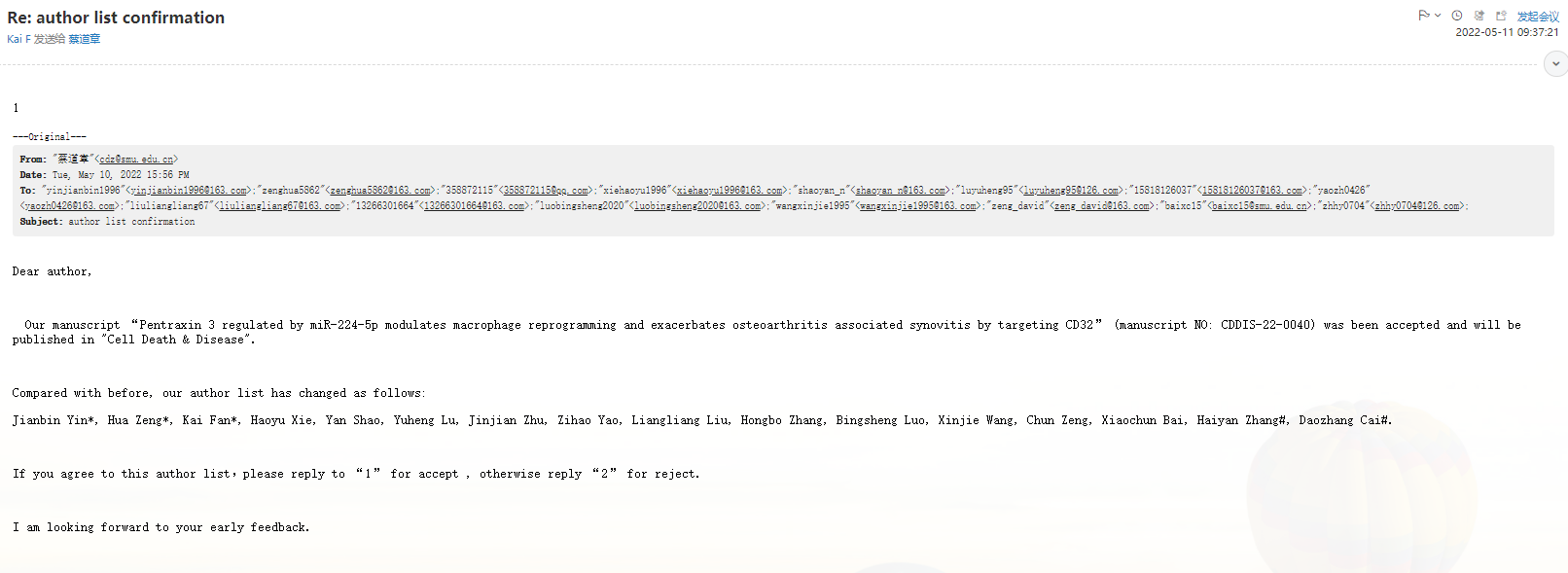


Dr. Haoyu Xie

[xiehaoyu1996@163.com](mailto:xiehaoyu1996@163.com)


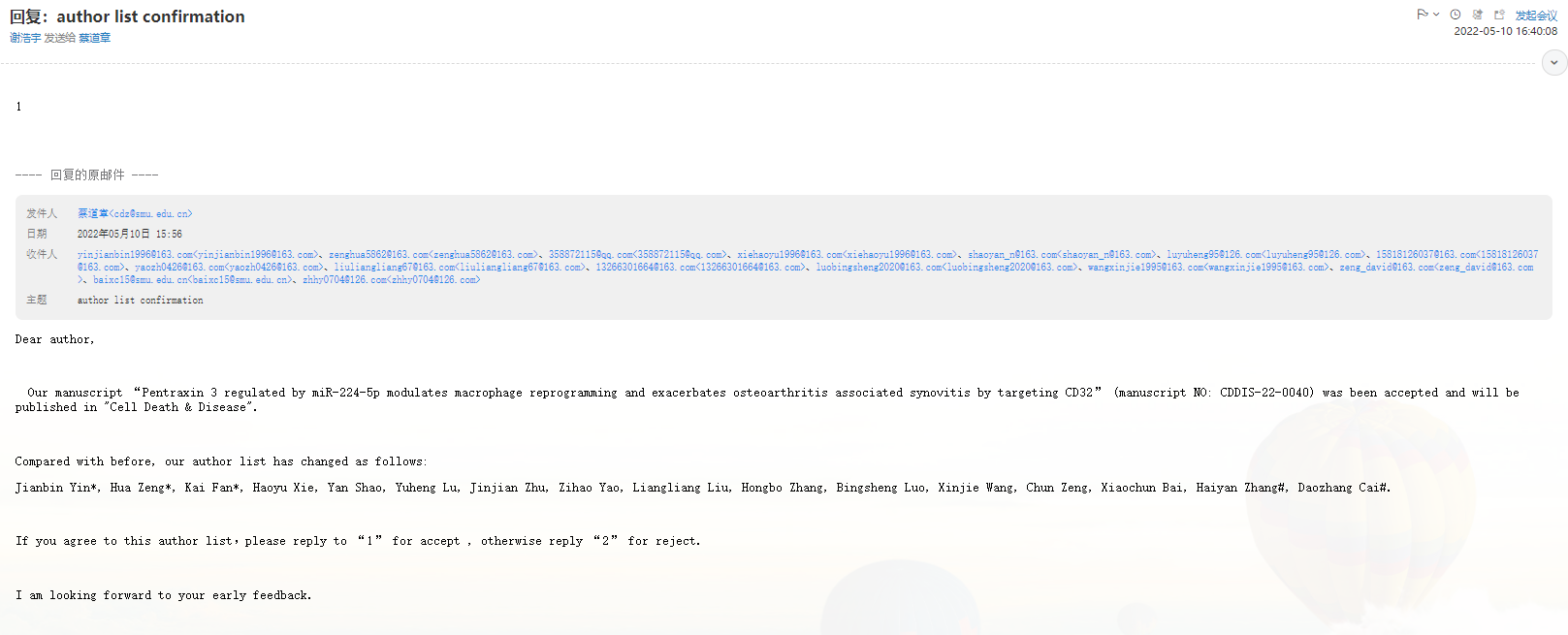


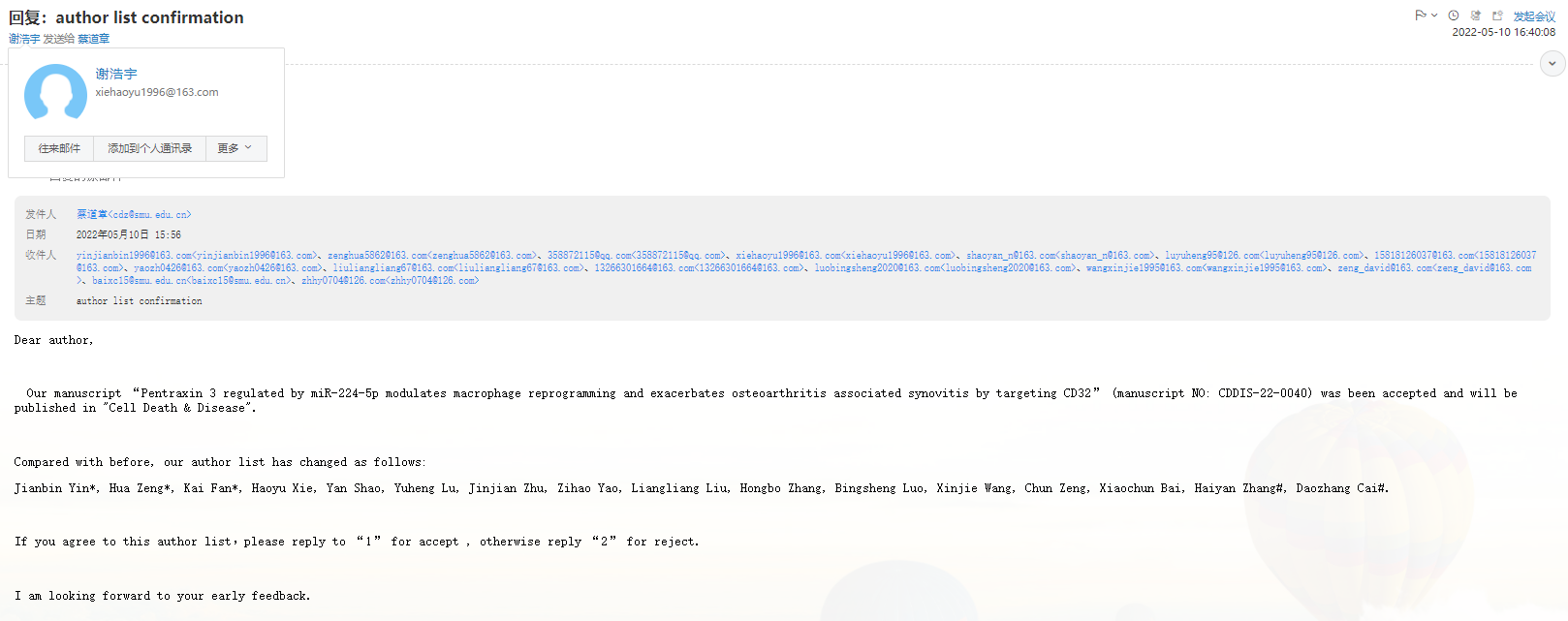


Dr. Yan Shao

[shaoyan_n@163.com](mailto:shaoyan_n@163.com)


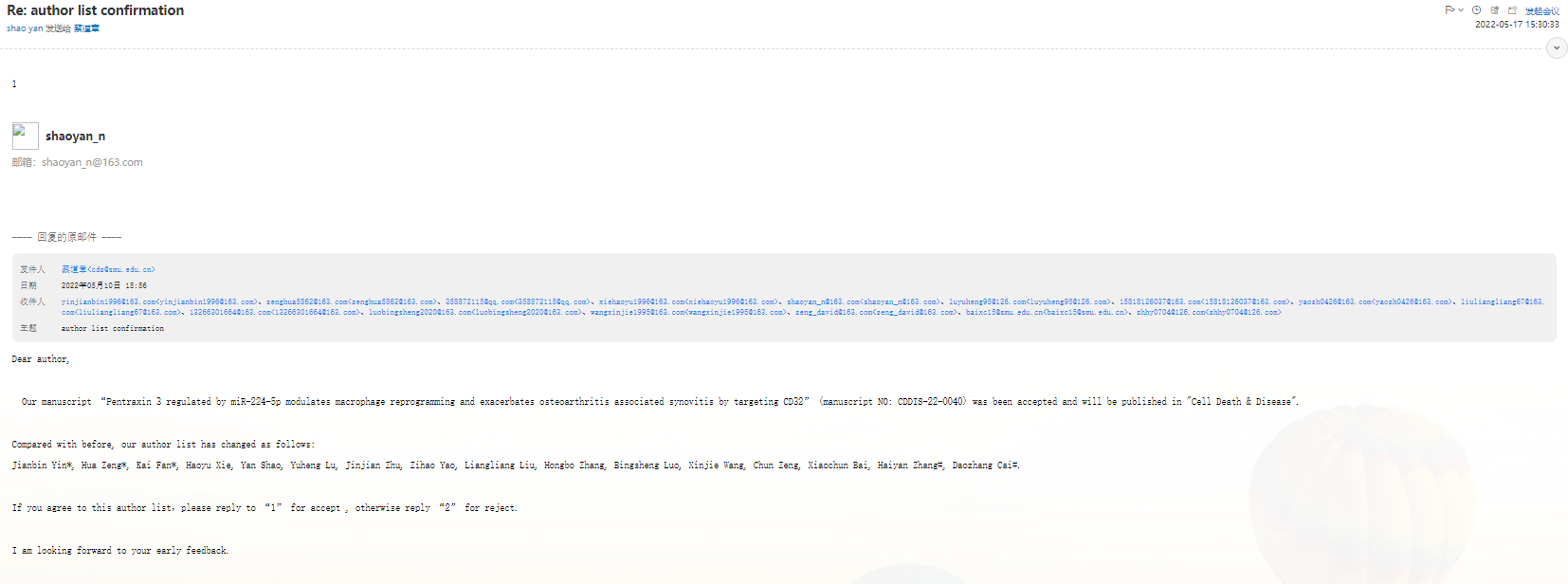


Dr. Yuheng Lu

[luyuheng95@126.com](mailto:luyuheng95@126.com)


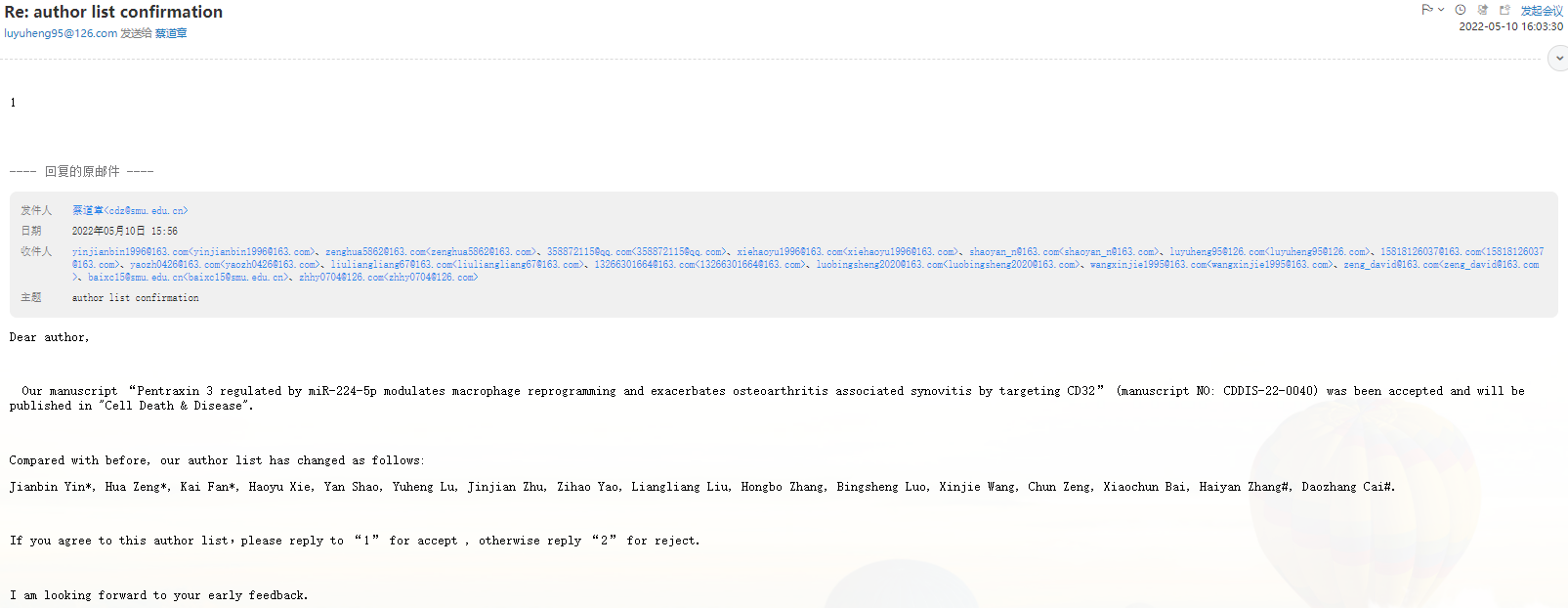


Mr. Jinjian Zhu

[15818126037@163.com](mailto:15818126037@163.com)


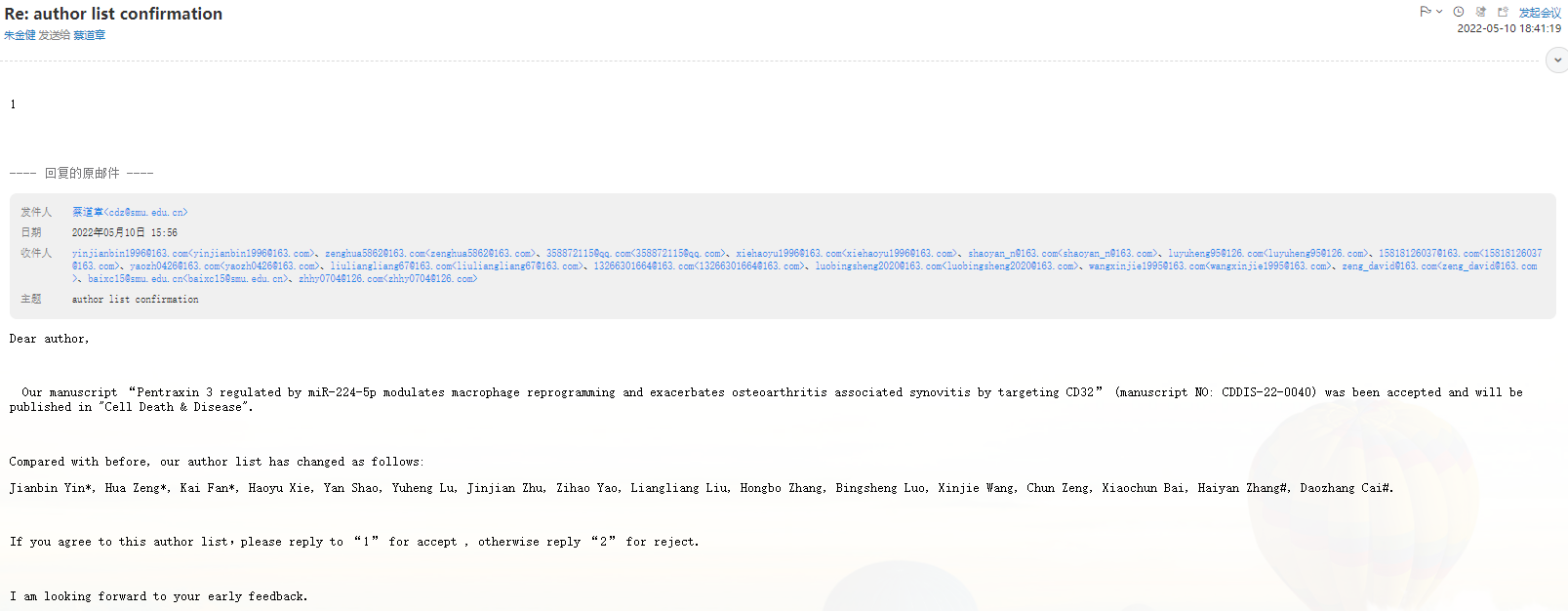


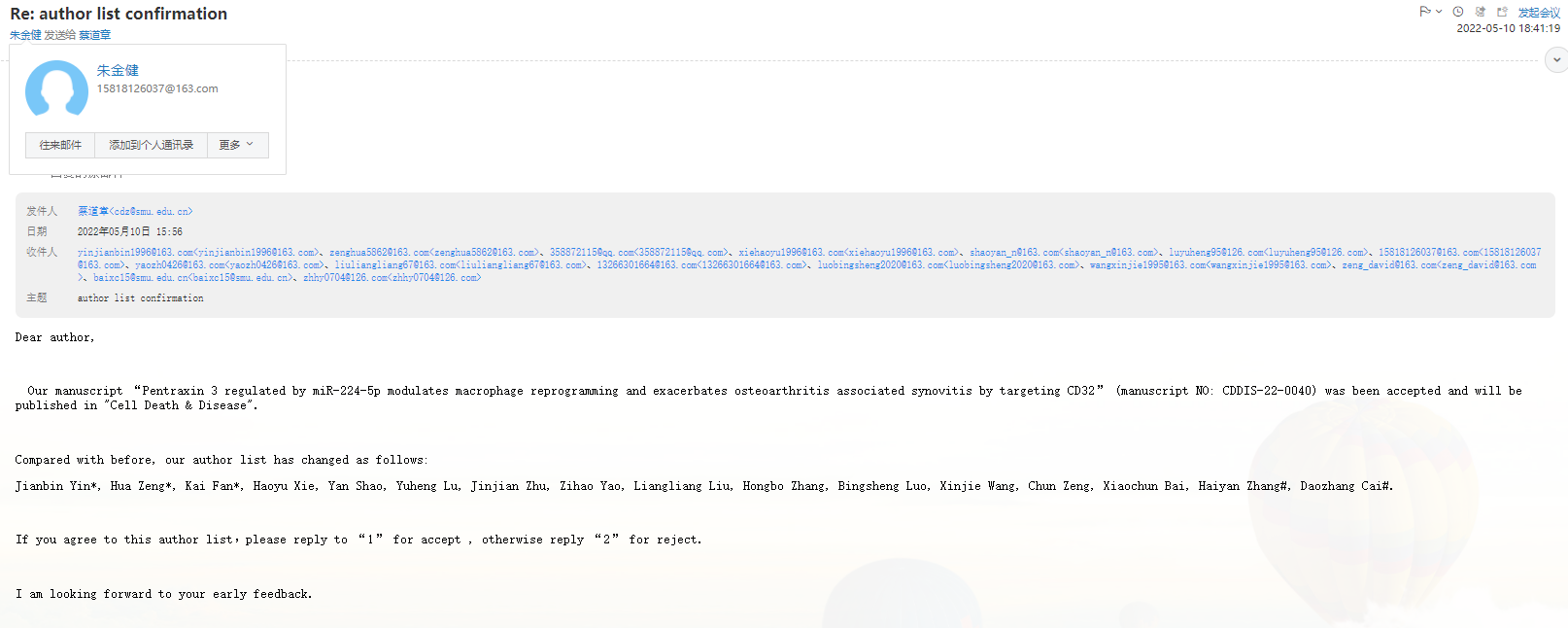


Dr. Zihao Yao

[yaozh0426@163.com](mailto:yaozh0426@163.com)


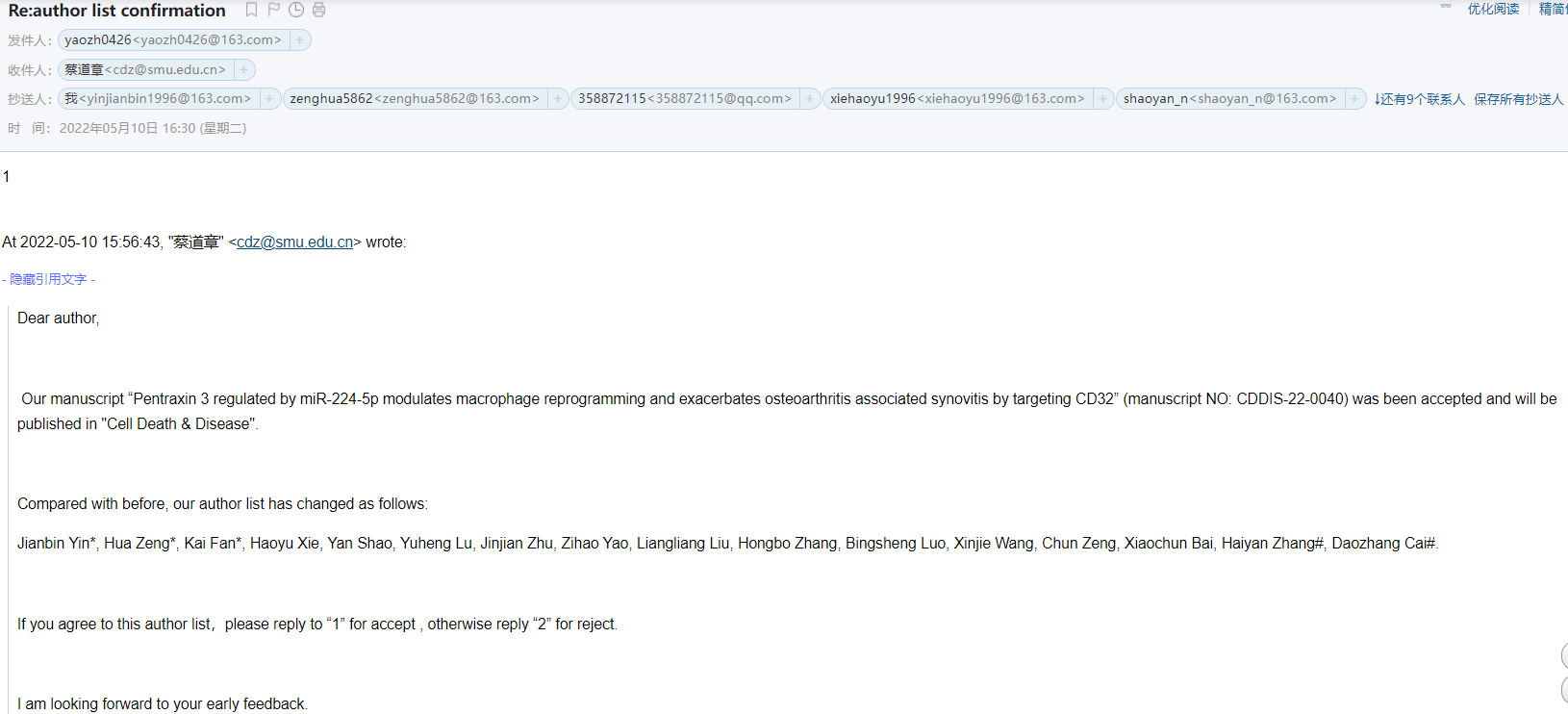


Dr. Liangliang Liu

[liuliangliang67@163.com](mailto:liuliangliang67@163.com)


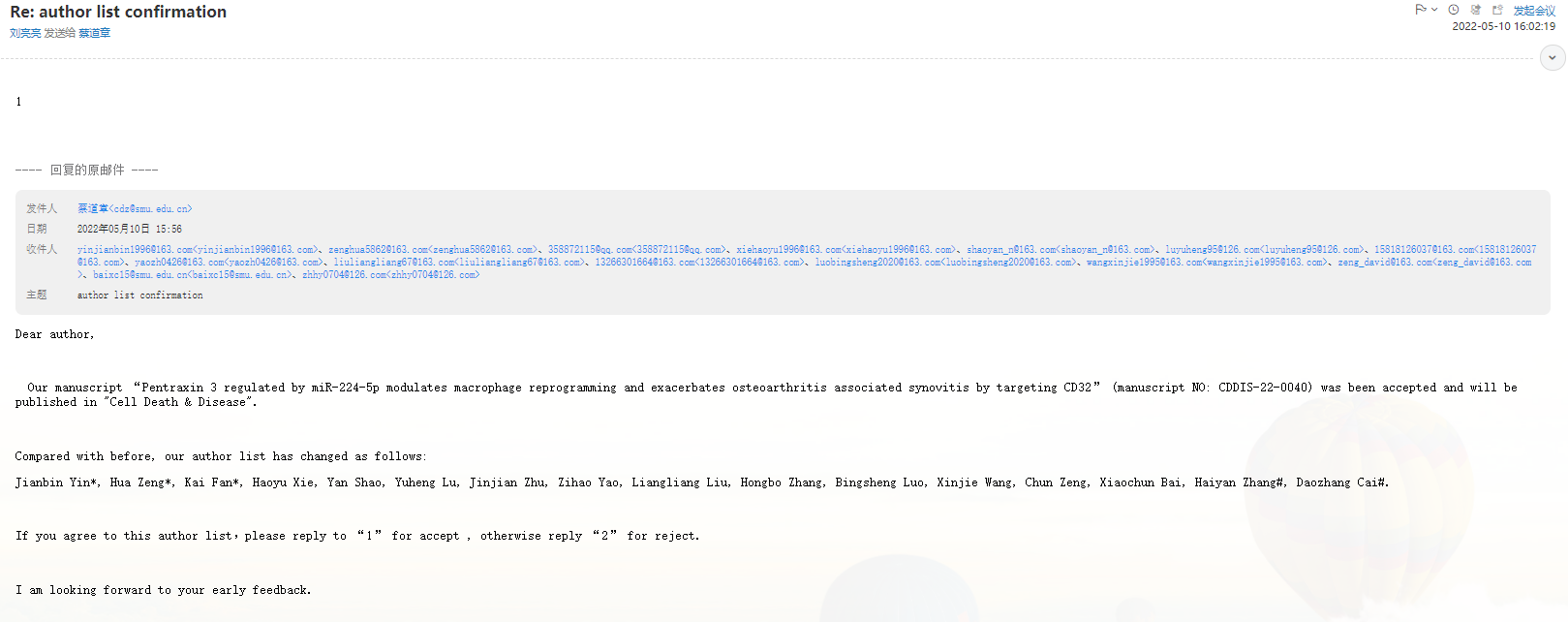


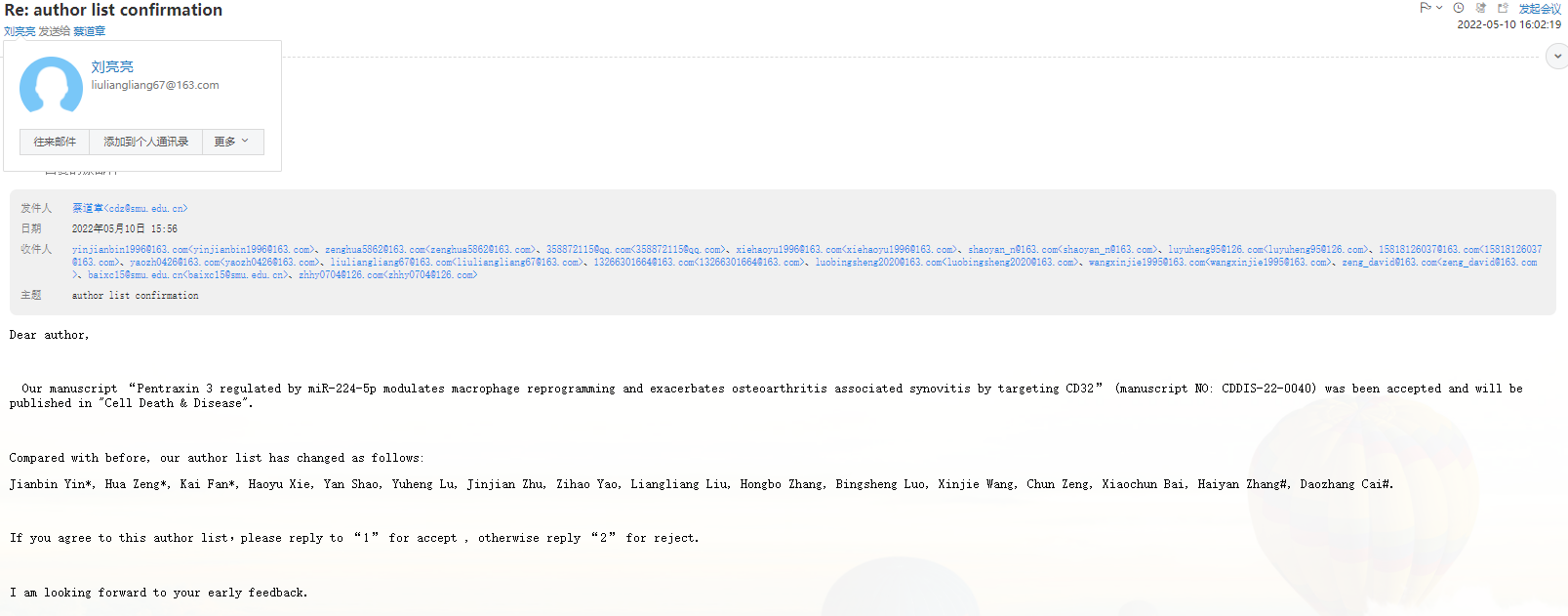


Dr. Hongbo Zhang

[13266301664@163.com](mailto:13266301664@163.com)


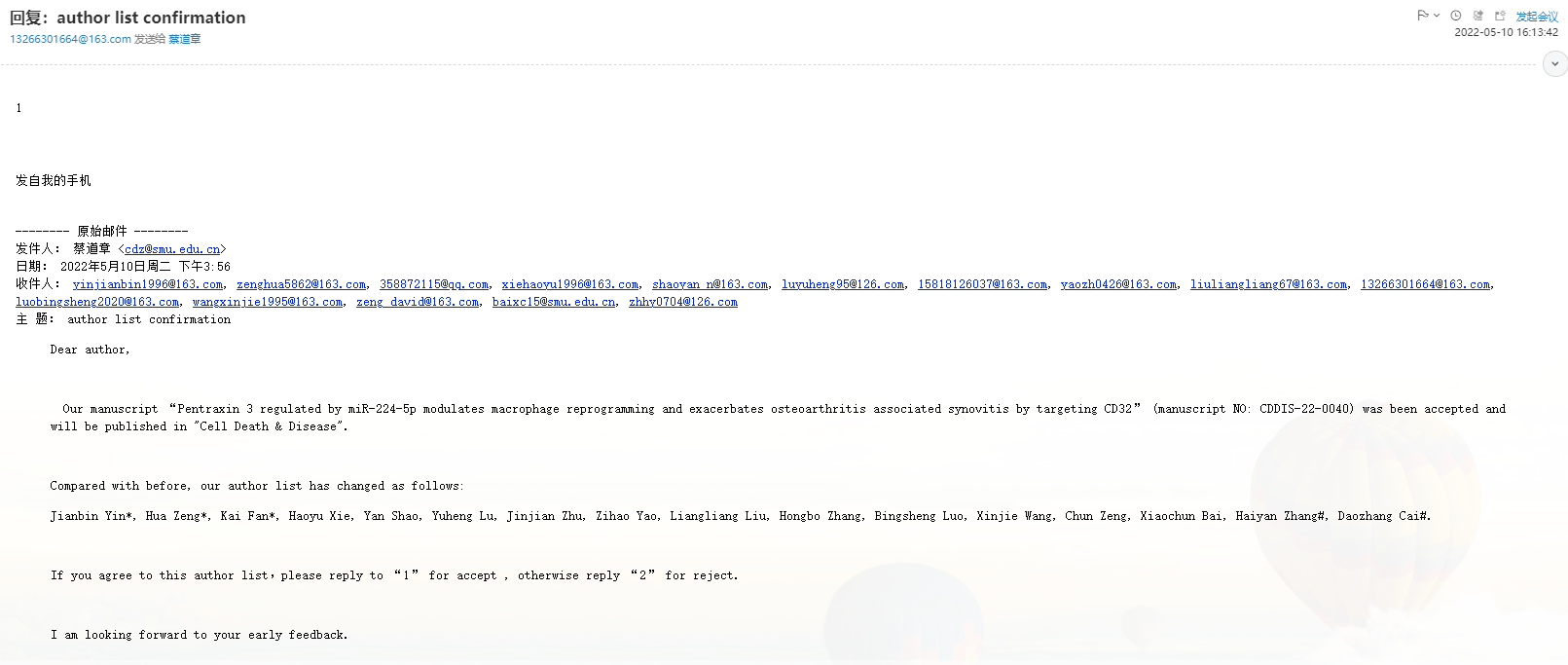


Dr. Bingsheng Luo

[luobingsheng2020@163.com](mailto:luobingsheng2020@163.com)


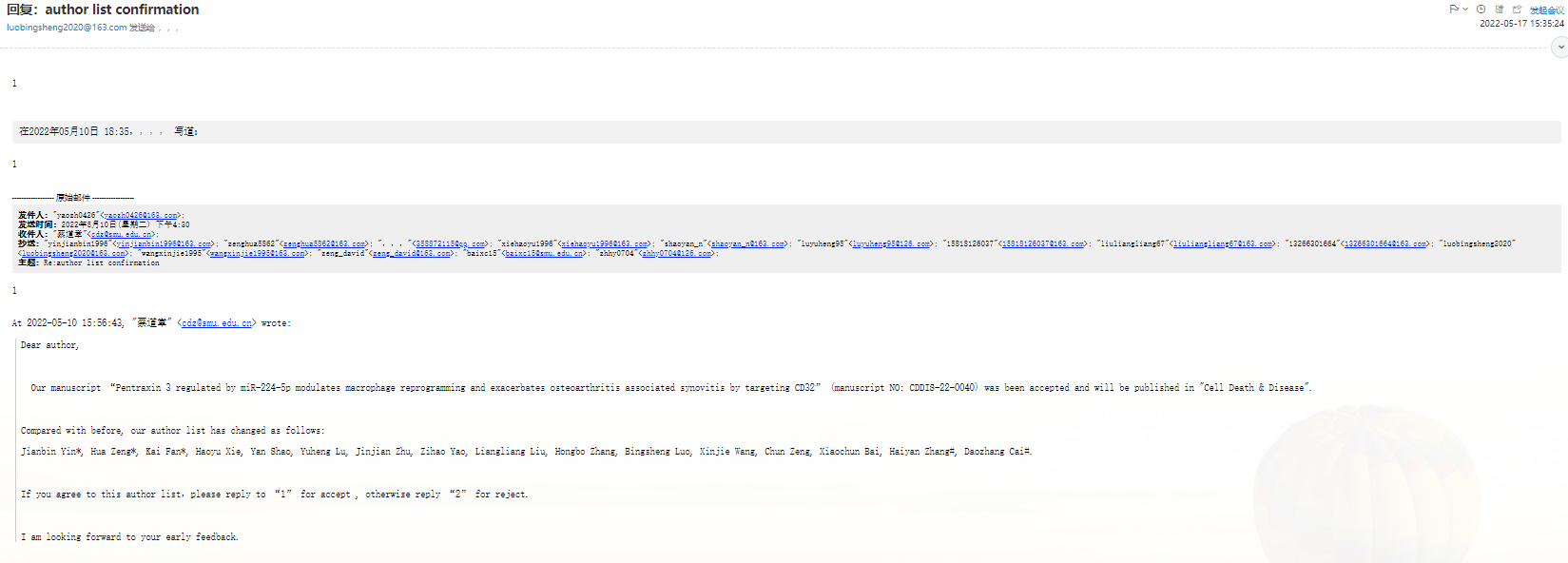


Mr. Xinjie Wang

wangxinjie1995@163.com


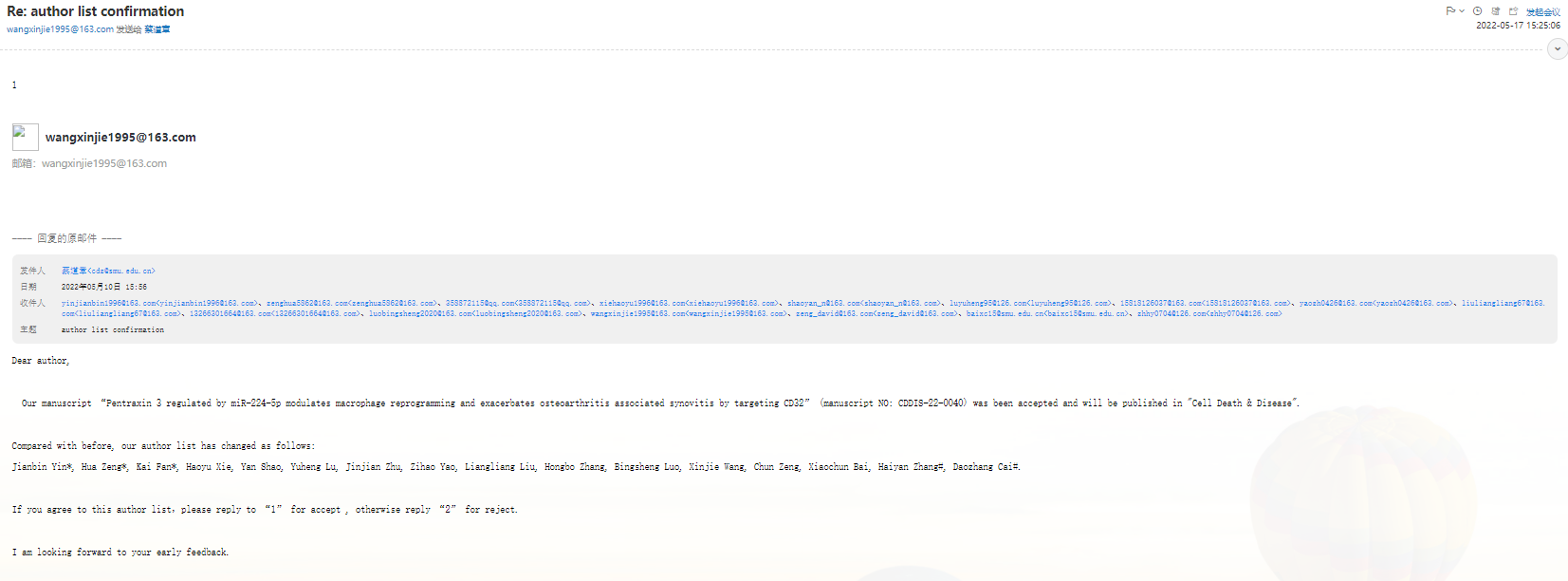


Professor Chun Zeng

zeng_david@163.com


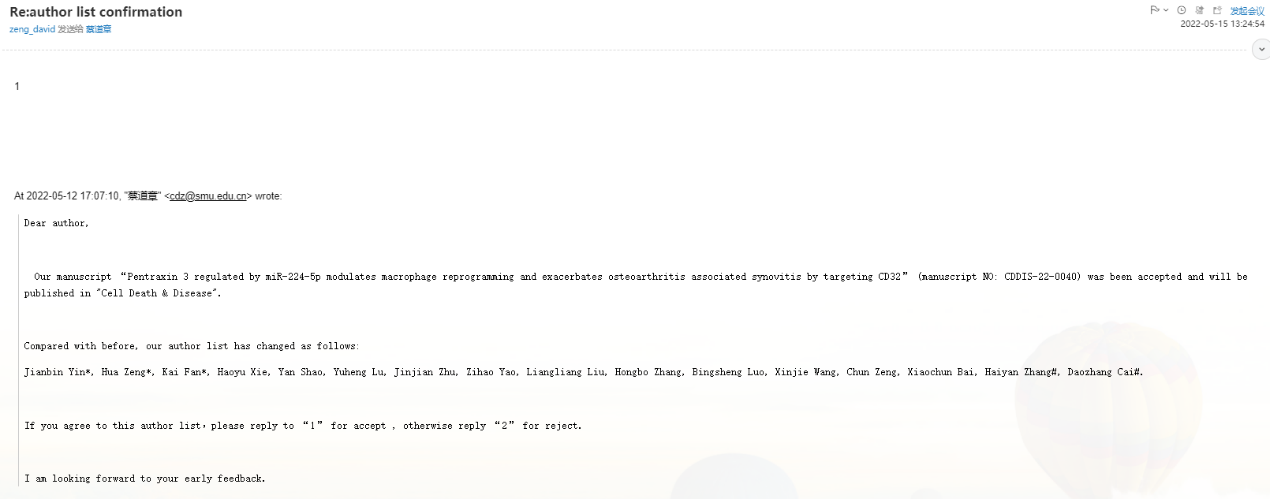


Professor Xiaochun Bai

[baixc15@smu.edu.cn](mailto:baixc15@smu.edu.cn)


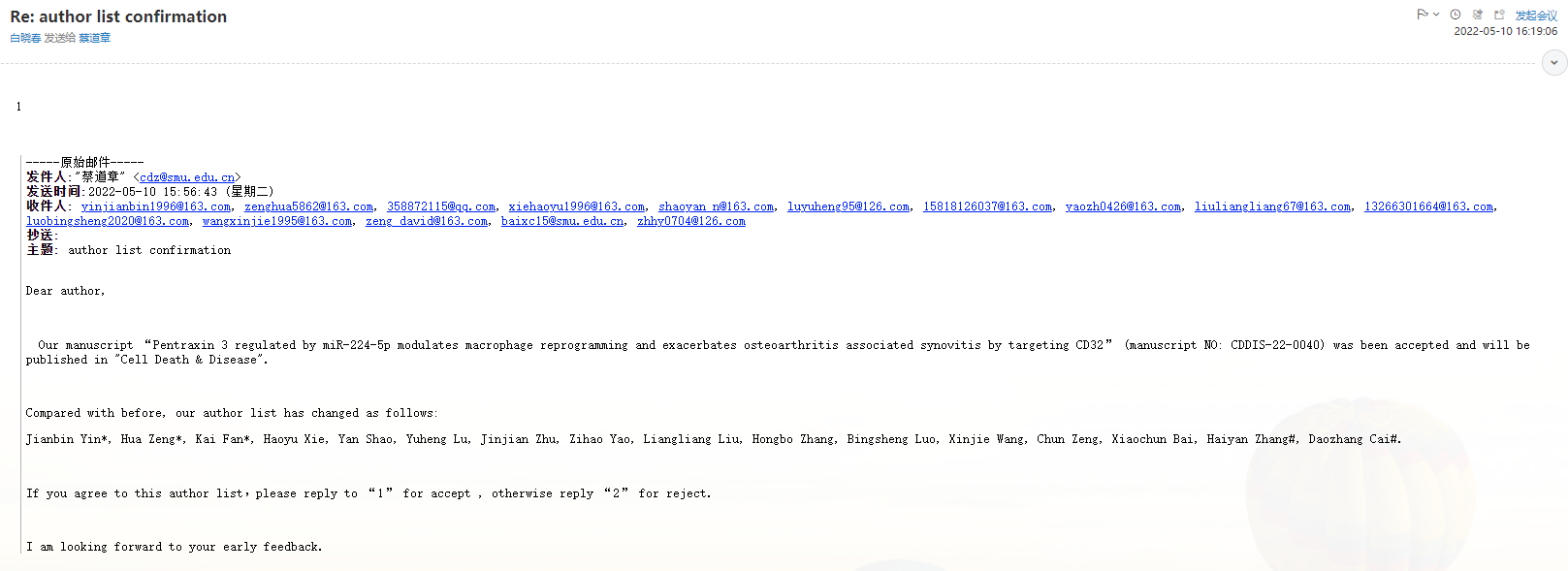


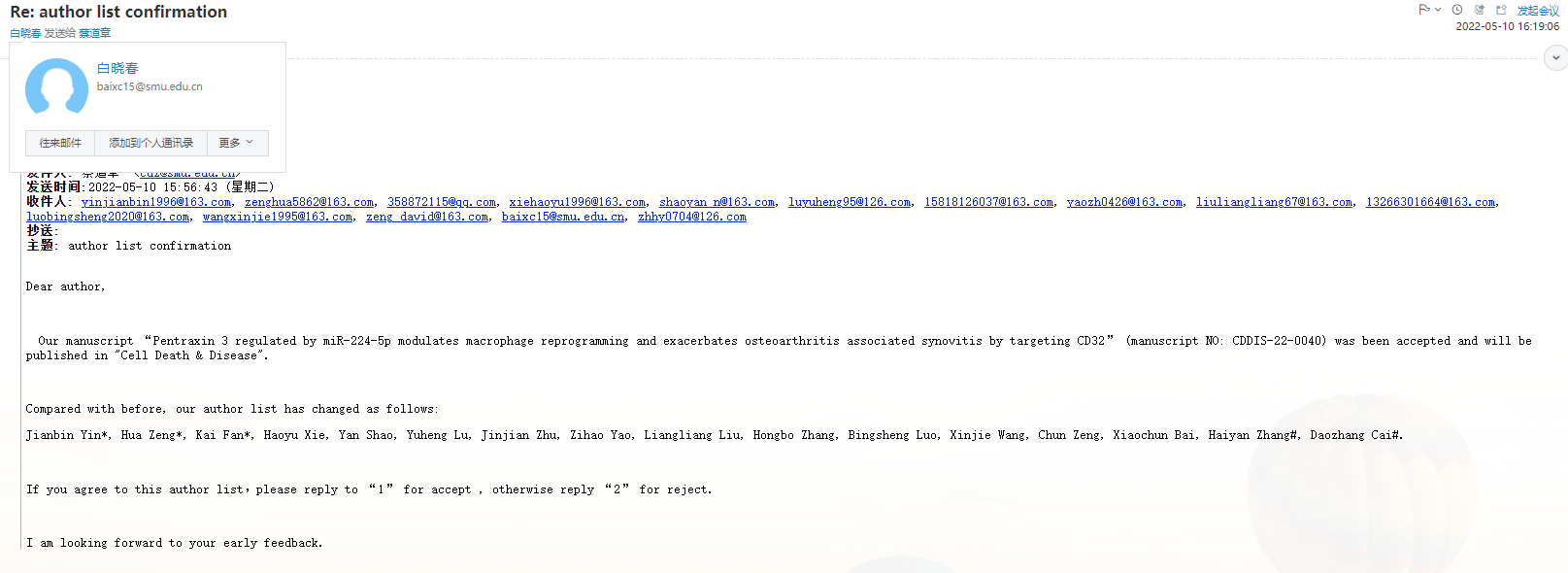


Dr. Haiyan Zhang

zhhy0704@126.com


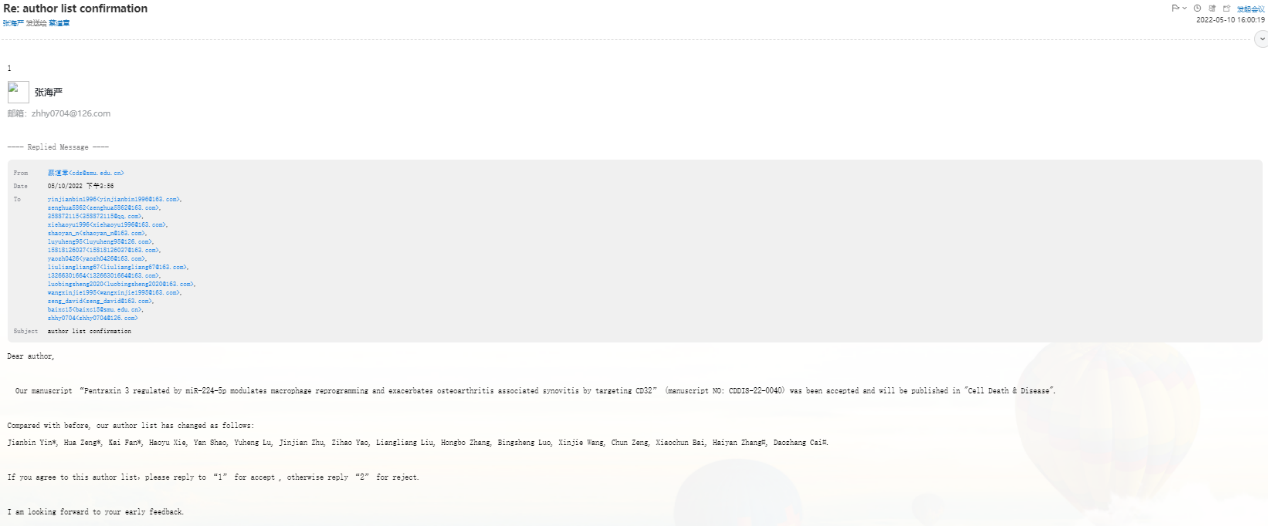

Supplement: Supplementary file 11 — Agreement from all authors about the author list [file 41419_2022_4962_MOESM11_ESM.docx]
